# Anaerobic fermentation featuring wheat bran and rice bran realizes the clean transformation of Chinese cabbage waste into livestock feed

**DOI:** 10.3389/fmicb.2023.1108047

**Published:** 2023-03-24

**Authors:** Jiawei Li, Cheng Wang, Shanshan Zhang, Jinxu Xing, Chunsheng Song, Qingwei Meng, Jianping Li, Shuo Jia, Anshan Shan

**Affiliations:** The Institute of Animal Nutrition, Northeast Agricultural University, Harbin, China

**Keywords:** Chinese cabbage waste, anaerobic fermentation, resource recycling, fermentation performance, bacteriome

## Abstract

Rapid aerobic decomposition and a high cost/benefit ratio restrain the transformation of Chinese cabbage waste into livestock feed. Herein, anaerobically co-fermenting Chinese cabbage waste with wheat bran and rice bran at different dry matter levels (250, 300, 350 g/kg fresh weight) was employed to achieve the effective and feasible clean transformation of Chinese cabbage waste, and the related microbiological mechanisms were revealed by high-throughput sequencing technology. The bran treatments caused an increase in pH value (4.75–77.25%) and free amino acid content (12.09–152.66%), but a reduction in lactic acid concentration (54.58–77.25%) and coliform bacteria counts (15.91–20.27%). In addition, the wheat bran treatment improved the levels of short-chain fatty acids, nonprotein nitrogen, water-soluble carbohydrates and antioxidant activity and reduced the ammonia nitrogen contents. In contrast, the rice bran treatment decreased the levels of acetic acid, water-soluble carbohydrates, nonprotein nitrogen, ammonia nitrogen, and antioxidant activities. Microbiologically, the bran treatments stimulated *Pediococcus*, *Lactobacillus*, *Enterobacter*, and *Weissella* but inhibited *Lactococcus* and *Leuconostoc*, which were the primary organic acid producers reflected by the redundancy analysis. In addition, Chinese cabbage waste fermented with wheat bran at 350 g/kg fresh weight or with rice bran at 300 g/kg fresh weight increased the scale and complexity of bacteriome, promoted commensalism or mutualism and upregulated the global metabolism pathways, including carbohydrate and amino acid metabolisms. Furthermore, the bran treatments resulted in an increase in bacterial communities that were facultatively anaerobic, biofilm-formed, Gram-negative, potentially pathogenic and stress-tolerant. Collectively, the bran treatments inhibited effluent formation and protein degradation and improved nutrient preservation but reduced organic acid production during the anaerobic fermentation, which is linked to the variations in the bacteriome, indicating that the constructed fermentation system should be further optimized.

## 1. Introduction

Demands for livestock feed restrain the development of animal husbandry. In the past years, the stagnant improvement of crop yield and terrestrial biodiversity loss caused by expanding agricultural land has encouraged researchers to develop massive, feasible and sustainable feed resources (Newbold et al., 2015). Chinese cabbage is cultivated worldwide and throughout the year, while the disposal of Chinese cabbage waste causes severe problems for environmental protection because of the yield scale and nutrient enrichment, highlighting that recycling this waste in an effective and eco-friendly method is essential for the environment and human health (Fausto-Castro et al., 2020). Papargyropoulou et al. (2014) demonstrated that transforming vegetable waste into livestock feed is a valorized and sustainable method. Therefore, without competing with human food supply chains, Chinese cabbage waste emerges as a plausibly sustainable feed resource (Lin et al., 2013).

Aerobic solid-state fermentation is a transformation technique to produce stabilized and high-protein feed ingredients based on vegetable and fruit discards (Ibarruri et al., 2021). However, the dried and sterilized procedure of aerobic solid-state fermentation significantly increases the cost/benefit ratio, and the cell wall components of the fungal inoculum improve the structural carbohydrate levels and decrease nutrient availability (Fausto-Castro et al., 2020; Ibarruri et al., 2021). In contrast, anaerobic fermentation, which acidifies materials by producing organic acid, might be preferred for recycling Chinese cabbage waste because the low pH value inhibits most undesired microorganisms and high organic acid levels promote the acid hydrolysis of structure carbohydrates, such as hemicellulose. In this context, the high moisture level is the main bottleneck in recycling Chinese cabbage waste, contributing to the effluent formation and soluble nutrient loss (Okine et al., 2007). Previous studies indicated that introducing water-holding materials into the fermentation system could be a feasible and cost-effective method for adjusting the undesired unsatisfactory moisture level, and a moisture level of 65–75% is preferred for anaerobic fermentation (Ren H. et al., 2019; Wang et al., 2019). Generally, the moisture level notably influences microbial metabolism resulting in a significant difference in fermentation performance (He et al., 2020b). Furthermore, the moisture level also impacts the growth of protein-degrading microbes, such as that *Clostridium* and *Enterobacter*, which are preferred in conditions with a high moisture level, causing severe protein hydrolysis and degradation, followed by the reduction in protein quality and alkaline pollutant generation (He et al., 2020d). However, variations in the epiphytic microbiome and different microbial succession lead to different responses to the exogenous and endogenous stimuli, highlighting that the preferred fermentation moisture levels and water-holding materials for recycling Chinese cabbage waste by anaerobic fermentation should be researched in detail under the microbiological context (Wang et al., 2020).

Cleaner production is a strategy that aims to create a production process with increased overall efficiency and reduced risks to humans and the environment (Santos et al., 2022). Furthermore, cleaner production could be classified into the following three levels: (1) waste reduction in its resource; (2) internal recycling actions; (3) waste reuse strategies such as external recycling and biogenic cycles (Goncales et al., 2018). Therefore, based on the third level of the concept of cleaner production, the present study employed wheat bran/rice bran as water-holding materials to counteract the high moisture level of Chinese cabbage waste and used three mixed mass ratios to optimize the fermentative system. Organic acid production, culturable microbial profiles, nitrogen distribution, chemical composition and antioxidant activity were employed to estimate fermentation characteristics, nutrient loss and alkaline pollutant production during anaerobic fermentation. High-throughput sequencing technology based on 16 S ribosomal RNA was used to analyze the variations in the dominated microbes, microbiome network topologies, predicted functional profiles, bacteriome phenotypes and correlation between microbes and environmental factors to evaluate the constructed microbiome and reveal the underlying microbiological mechanism.

## 2. Materials and methods

### 2.1. Materials preparation

Chinese cabbage waste was collected from the market in Harbin, Heilongjiang Province, China. Subsequently, Chinese cabbage waste was chopped 1–2 cm in length. Before mixing, fresh Chinese cabbage waste, wheat bran, and rice bran were sampled for later analysis and then randomly divided into different groups according to the Editorial Note of design principles of experiments (Robinson et al., 2006). Ren H. et al. (2019) indicated that a dry matter (DM) level of 250–350 g/kg fresh weight (FW) was suitable for anaerobic fermentation, whereas He et al. (2020a) demonstrated that a DM level of 300–350 g/kg FW contributed to the anaerobic fermentation. Therefore, in the present study, Chinese cabbage waste was mixed with wheat bran at a mass ratio of 383:117 (W1), 353:147 (W2), and 323:177 (W3), resulting in a DM content of 250, 300, and 350 g/kg FW, respectively. Chinese cabbage waste was mixed with rice bran at a mass ratio of 387:113 (R1), 358:142 (R2), and 329:171 (R3), resulting in a DM content of 250, 300, and 350 g/kg FW, respectively. Chinese cabbage waste mixed without wheat bran/rice bran was the control group (Con). After thoroughly mixing by hand, 500 g of materials were packed into polyethylene bags (20 cm × 30 cm) and sealed with a vacuum sealer. The bags were stored at room temperature (20–25 degrees celsius), and a triplicate silo per group was sampled on days 1, 3, 5, 7, 15, 30, and 60. Thus, 147 bags (7 time durations × 7 treatments × 3 replicates) were employed for the fermentation experiment.

### 2.2. Effluent, chemical composition, and fermentation characteristics

A graduated cylinder was used to determine the amount of effluent. Samples were dried in an oven at 65 degrees celsius for 72 h to determine the DM content and then milled to pass a 1 mm screen with a laboratory mill. The total nitrogen (TN) was determined by a Kjeldahl nitrogen analyzer according to the Association of Official Analytical Chemists (AOAC) procedure, and the crude protein content was calculated as TN × 6.25 (AOAC, 1990). The neutral detergent fiber (NDF) and acid detergent fiber (ADF) contents were determined by an Ankom 220 fiber analyzer according to the method of Van Soest et al. (1991). The water-soluble carbohydrate (WSC) was determined with an anthrone reagent (Murphy, 1958).

Twenty grams of samples mixed with 180 ml of deionized water were blended for 30 s with a commercial blender and then filtered with 4 layers of cheesecloth and filter paper. The filtered liquor was used to determine the pH value with a glass electrode pH meter (Sartorius, Göttingen, Germany). After centrifuging 18,000 × *g* for 15 min, the liquid supernatant was collected and used to determine the organic acid concentration by gas chromatography (GC-2010, SHIMADZU, Japan) fitted with a fused-silica capillary column (DB-FATWAX, Agilent). The lactic acid content was measured using high-performance liquid chromatography with a C18 column at 30 degrees celsius (Li J. et al., 2022).

### 2.3. Culturable microbial profiles assay

Ten grams of samples were shaken with 90 ml of sterile sodium chloride solution (0.85%) at 150 rpm for half an hour. Subsequently, bacterial suspension was serially diluted, and 50 μl of suspension was sprayed on different agar plates to determine the counts of lactic acid bacteria (LAB; MRS, hopebio, Qingdao, China), coliform bacteria (Violet Red Bile Glucose Agar plate, hopebio, Qingdao, China) and yeast (Potato Dextrose Agar plate with chloramphenicol at 0.1 g/L, hopebio, Qingdao, China).

### 2.4. Antioxidant assay

Briefly, 0.2 g of lyophilized samples were extracted with 10 ml of methanol for 24 h. Then, the suspensions were centrifuged at 18,000 × *g* for 10 min, and the supernatant was employed as the crude extract solution.

2,2-diphenyl-1-picrylhydrazyl (DPPH) assay was performed as described previously (He et al., 2019b). Briefly, 0.1 mM DPPH (methanol solution) was incubated with the crude enzyme solution in a dark place at room temperature for 30 min. Trolox solution with various concentrations was employed as the standard solution. After incubation, the absorbance of the solution was read at 517 nm.

2,2-azinobis-3-ethylbenzothiazoline-6-sulfonic acid diammonium salt radical cation (ABTS) assay was performed as described previously (He et al., 2019b). Briefly, 7 mM ABTS solution (water solution) was incubated with equal volumes of 2.45 mM potassium persulfate solution for 12–16 h at room temperature to obtain the ABTS^+^ solution. Then, the ABTS^+^ solution was diluted 10-fold with methanol and incubated with the crude enzyme solution for 10 min. Trolox solution with various concentrations was employed as the standard solution. After incubation, the absorbance of the solution was read at 734 nm.

The ferric-reducing antioxidant power (FRAP) assay was performed as described previously (He et al., 2019b). The sodium acetate solution (pH 3.6) was mixed with 10 mM 2,4,6-tripyridy-s-triazine solution and 20 mM FeCl_3_ solution at a volume ratio of 10:1:1 to obtain the working solution. Then, the working solution was incubated with the crude enzyme solution at 37 degrees celsius for 30 min. Trolox solution with various concentrations was employed as the standard solution. After incubation, the absorbance of the solution was read at 593 nm.

### 2.5. Nitrogen distribution assay

The dried samples were mixed with the distilled and trichloracetic acid solution (10% m/v) for 30 min, and then liquid parts were removed, and the residue was employed to determine the nonprotein nitrogen (nonprotein-N) according to the method of TN (Licitra et al., 1996). Ten ml of trichloroacetic acid solution (25% m/v) was added to 40 ml of filter liquor and then incubated for 1 h to precipitate true protein. After centrifuging 18,000 × *g* for 15 min, the supernatant was used to determine ammonia-N and free amino acid-N contents (Broderick and Kang, 1980).

### 2.6. DNA extraction, quality control, clustering, taxonomy annotation, and bioinformatics analysis

According to the manufacturer’s instructions, the total DNA was extracted with the E.Z.N.A. stool DNA kit (Omega Biotek, Norcross, GA, US). The V3-V4 region of the 16S rDNA gene was amplified using primers 341F (5′-CCTACGGGNGGCWGCAG) and 806R (5′-GGACTACHVGGGTATCTAAT). According to the standard protocols, the amplified DNA was purified and then sequenced by Guangzhou Gene Denovo Co. Ltd. (Guangzhou, China) using the Illumina platform. The raw data were filtered using the FASTP (version 0.18.0), merged as raw tags using FLASH (version 1.2.11) and then filtered to obtain the high-quality clean tags. The clean tags were clustered into operational taxonomic units (OTUs) of 97% similarity according to UPARSE (version 9.2.64) pipeline. The chimeric tags were removed according to the UCHIME algorithm to get effective tags. The RDP classifier classified the representative OTU sequences into organisms by a naive Bayesian model based on the SILVA database.

The principal coordinate analysis (PCoA) was performed using the Vegan package in the R project. Venn analysis and upset plot were performed in the R project VennDiagram package and UpSetR package, respectively. The linear discriminant analysis effect size (Lefse) was performed by Lefse software (version 1.0). Redundancy analysis (RDA) and Variation partition analysis (VPA) were executed in the R project Vegan package (version 2.5.3). The co-occurrence network was analyzed by Spearman correlation analysis with an absolute value >0.8 and *p* value < 0.05. The PICRUSt (version 2.1.4) was used in the KEGG pathway analysis of OTUs. The bacteriome phenotypes were classified using BugBase. Tukey’s HSD test was performed using the Vegan package in the R project.

### 2.7. Statistical analysis

Data of fermentative characteristics, nitrogen distribution, and microbial profiles during the whole process were analyzed by two-way ANOVA using SPSS 22.0 software (IBM, Chicago, IL, United States) with the model: Y_ij_ = μ + α_i_ + β_j_ + (α × β)_ij_ + e_ij_, where Y_ij_ is the dependent variable; μ is the overall mean; α_i_ is the fixed effect of the wheat bran/rice bran treatment; β_j_ is the fixed effect of time duration; (α × β)_ij_ is the interaction between the bran treatment and time duration and e_ij_ is the residual error. The data on antioxidant activities and chemical composition by one-way ANOVA. Tukey’s test was employed for multiple comparisons. The differences were defined as significant when the *p* value was less than 0.05.

## 3. Results

### 3.1. Effluent yield and fermentation parameters during anaerobic fermentation

**Figure 1:**
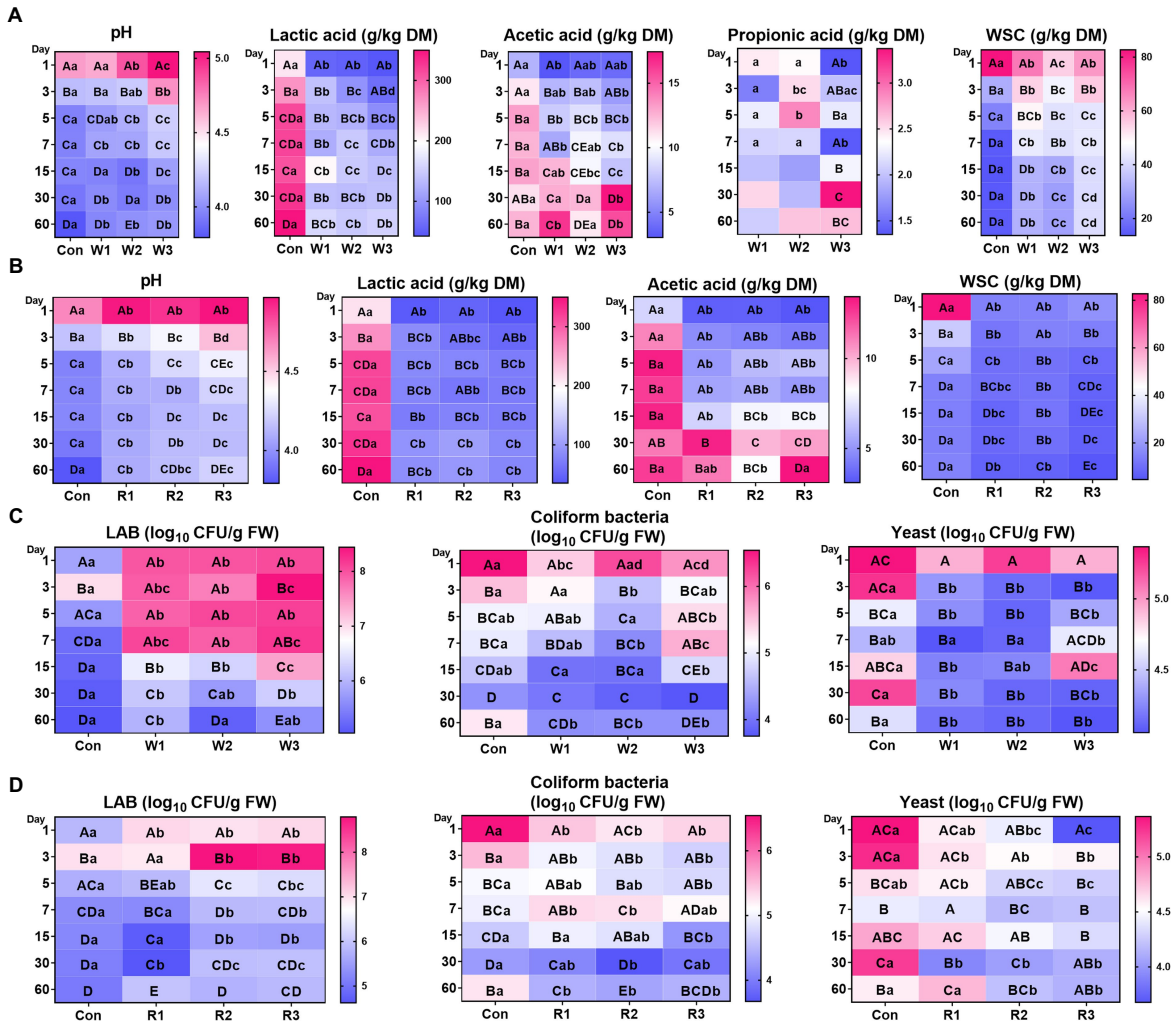
Fermentative characteristics **(A,B)** and culturable microbial profiles **(C,D)** during the anaerobic fermentation of Chinese cabbage waste. The control group (Con). Chinese cabbage waste was mixed with wheat bran at a mass ratio of 383:117 (W1), 353:147 (W2), and 323:177 (W3) or with rice bran at 387:113 (R1), 358:142 (R2), and 329:171 (R3), respectively; DM, dry matter; WSC, water-soluble carbohydrate; FW, fresh weight; LAB, lactic acid bacteria; CFU, colony-forming unit. The significant difference (*p* < 0.05) between different days (column) in the same group is represented by the different capital letters; The significant difference (*p* < 0.05) between different groups (row) on the same day is represented by the different lowercase letters.

Supplemental Table S1 showed rapid effluent formation in Con from days 1–3, whereas no effluent formation was found in the bran treatment groups. An interaction between time duration and bran introduction was observed for all the tested parameters (Figures 1A,B; Supplemental Table S1). The bran treatments significantly decreased the pH value and lactic acid levels compared with Con, whereas no significant difference in pH values between W1 and Con was observed from days 1–5. In addition, most of the bran treatment groups exhibited lower (*p* < 0.05) acetic acid levels than Con from days 1–15. In contrast, except for R2, no significant difference in acetic acid levels was observed between the bran treatment groups and Con from days 30–60, and W1 and W3 exhibited a higher (*p* < 0.05) acetic acid level than Con on day 60. Moreover, the wheat bran treatment significantly decreased the WSC content compared with Con on day 1, whereas it significantly increased the WSC content compared with Con from days 3–60. In contrast, the rice bran treatment significantly decreased the WSC content compared with Con from days 1–60. Furthermore, among the wheat bran treatment groups, W3 exhibited the highest (*p* < 0.05) pH value and WSC contents and the lowest (*p* < 0.05) lactic acid levels. In contrast, among the rice bran treatment groups, R3 exhibited the highest (*p* < 0.05) pH value and lowest (*p* < 0.05) levels of lactic acid and WSC.

### 3.2. Microbial cultivation profiles

An interaction between bran treatment and time duration was observed for all the tested microbial counts (Figures 1C,D; Supplemental Table S2). The bran treatment groups, except for R1, significantly increased the LAB counts compared with Con from days 1–30. R1 exhibited no significant difference from days 3–15 and lower (*p* < 0.05) LAB counts on day 30 than Con. In contrast, on day 60, only W1 exhibited higher (*p* < 0.05) LAB counts than Con. In addition, at most of the time duration, the bran treatments significantly decreased the counts of coliform bacteria and yeast. In the wheat bran treatment groups, W3 exhibited higher (*p* < 0.05) coliform bacteria count on day 7 and higher (*p* < 0.05) yeast counts on day 15 than Con. In the rice bran treatment groups, R1 and R2 exhibited higher (*p* < 0.05) coliform bacteria counts than Con on day 7. Furthermore, among the wheat bran treatment groups, W3 exhibited the highest (*p* < 0.05) counts of LAB, coliform bacteria and yeast. In contrast, R2 and R3 exhibited higher (*p* < 0.05) counts of LAB and lower (*p* < 0.05) counts of coliform bacteria and yeast than R1.

### 3.3. Nitrogen distribution

**Figure 2:**
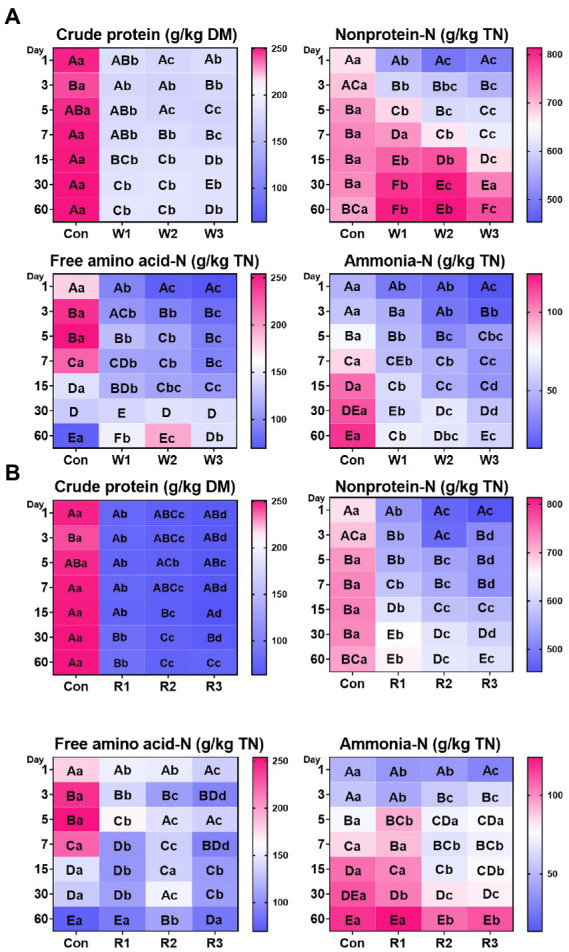
Nitrogen distribution **(A,B)** during the anaerobic fermentation of Chinese cabbage waste. The control group (Con). Chinese cabbage waste was mixed with wheat bran at a mass ratio of 383:117 (W1), 353:147 (W2), and 323:177 (W3) or with rice bran at 387:113 (R1), 358:142 (R2), and 329:171 (R3), respectively; TN, total nitrogen; The significant difference (*p* < 0.05) between different days (column) in the same group is represented by the different capital letters; The significant difference (*p* < 0.05) between different groups (row) in the same day is represented by the different lowercase letters.

An interaction between bran treatments and time duration was observed for all the tested indices (Figure 2; Supplemental Table S3). The bran treatments significantly decreased the crude protein levels compared with Con from days 1–60. In addition, the wheat bran treatment significantly decreased the content of nonprotein-N and free amino acid-N compared with Con from days 1–5, whereas it significantly increased the contents of nonprotein-N and free amino acid-N compared with Con on day 60. In contrast, the rice bran treatment significantly decreased nonprotein-N content from days 1–60 and free amino acid-N content from days 1–15 compared with Con, whereas R2 significantly increased the free amino acid-N content compared with Con on day 60. Moreover, most of the time, the wheat bran treatment significantly decreased the ammonia-N contents compared with Con. Among the rice bran treatment groups, R2 and R3 exhibited higher (*p* < 0.05) ammonia-N content on day 3, and R1 exhibited higher (*p* < 0.05) ammonia-N content on day 5 than Con. Furthermore, among the wheat bran treatment groups, W3 exhibited the lowest (*p* < 0.05) levels of nonprotein-N, free amino acid and ammonia-N. Among the rice bran treatment groups, R2 and R3 showed lower (*p* < 0.05) contents of nonprotein-N and ammonia-N than R1.

### 3.4. Chemical composition and antioxidant activity

**Figure 3:**
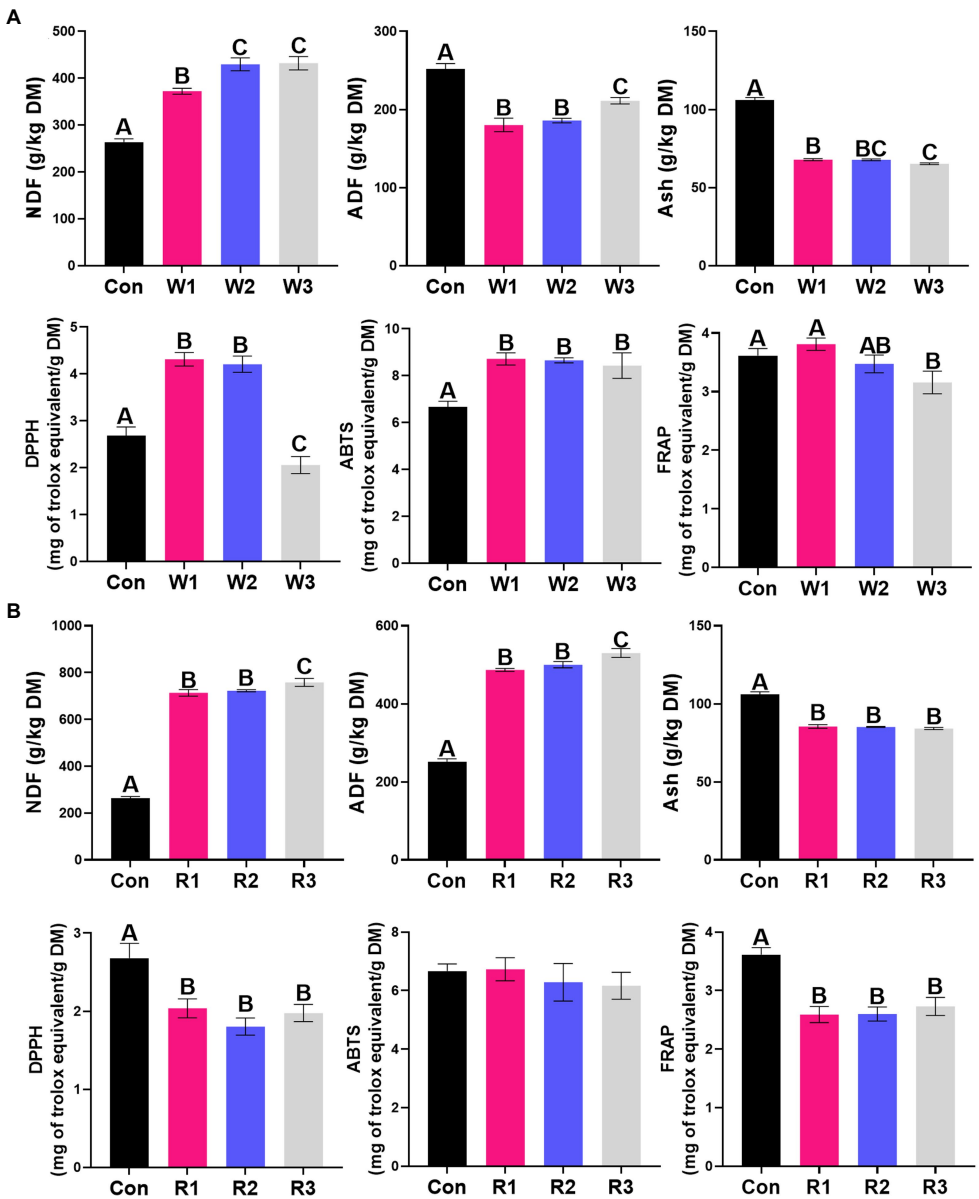
Chemical composition and antioxidant activity **(A,B)** during the anaerobic fermentation of Chinese cabbage waste. The control group (Con). Chinese cabbage waste was mixed with wheat bran at a mass ratio of 383:117 (W1), 353:147 (W2), and 323:177 (W3) or with rice bran at 387:113 (R1), 358:142 (R2), and 329:171 (R3), respectively; NDF, neutral detergent fiber; ADF, acid detergent fiber; Ash, crude ash; DPPH, free radical DPPH scavenging activity; ABTS, radical ABTS scavenging activity; FRAP, ferric reducing antioxidant power. The significant difference (*p* < 0.05) between different groups is represented by the different capital letters.

The bran treatment significantly influenced all the tested parameters except for the rice bran treatment, which did not significantly affect the ABTS activity (Figure 3; Supplemental Table S4). W1 and W2 exhibited higher (*p* < 0.05) DPPH and ABTS activities than Con, whereas W3 exhibited lower (*p* < 0.05) activities of DPPH and FRAP but higher (*p* < 0.05) ABTS activity than Con. In contrast, the rice bran treatment significantly decreased the activities of DPPH and FRAP compared with Con. The wheat bran treatment significantly increased the NDF content and decreased the ADF and Ash contents compared with Con. In contrast, the rice bran treatment significantly increased the contents of NDF and ADF but significantly decreased the Ash content compared with Con.

### 3.5. Microbial community composition

**Figure 4:**
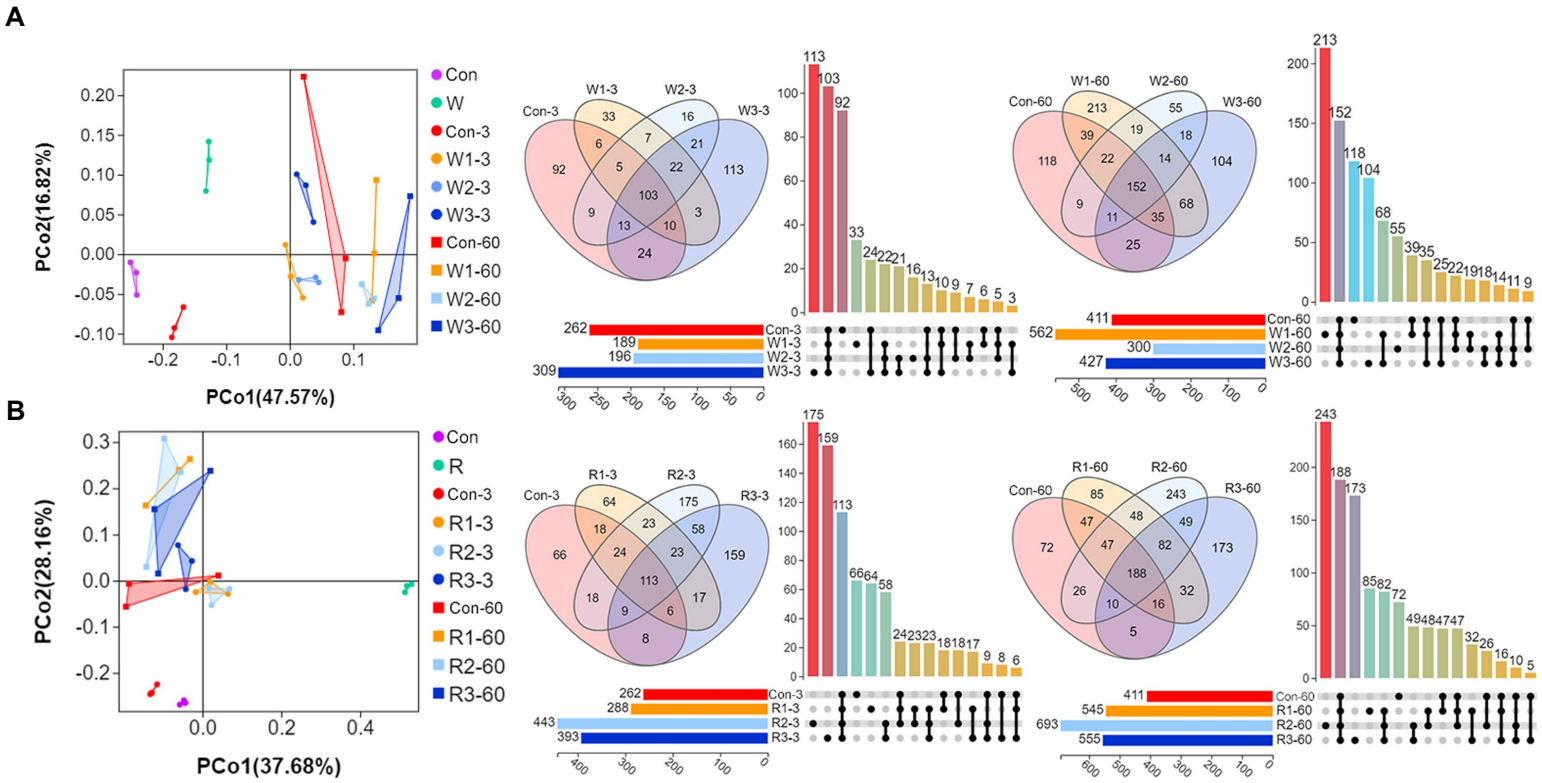
PCoA, Venn diagrams and UpSet plot of bacteriome during the anaerobic fermentation of Chinese cabbage waste. The control group (Con). Chinese cabbage waste was mixed with **(A)** wheat bran at a mass ratio of 383:117 (W1), 353:147 (W2), and 323:177 (W3) or with **(B)** rice bran at 387:113 (R1), 358:142 (R2), and 329:171 (R3), respectively; 3 and 60 mean day 3 and day 60, respectively.

Figure 4 shows a remarkably (Adonis test, *p* < 0.05) spatial difference between Con and bran treatment groups was observed. However, two-dimensional overlaps were observed within the wheat bran and rice bran treatment groups. Furthermore, Venn diagrams indicated that core and unique OTU counts increased with the fermentation process except for W3, wherein unique OTU counts decreased to 103 on day 60.

**Figure 5:**
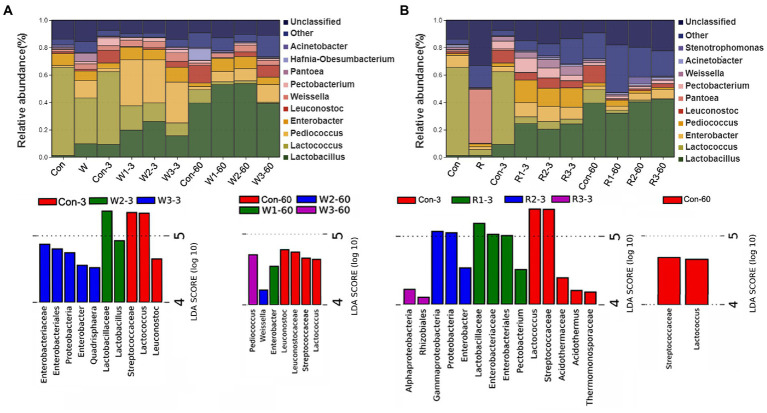
Microbial community composition and Lefse of bacteriome during the anaerobic fermentation of Chinese cabbage waste. The control group (Con). Chinese cabbage waste was mixed with **(A)** wheat bran at a mass ratio of 383:117 (W1), 353:147 (W2), and 323:177 (W3) or with **(B)** rice bran at 387:113 (R1), 358:142 (R2), and 329:171 (R3), respectively; 3 and 60 mean day 3 and day 60, respectively.

The bacterial communities at the genus level are shown in Figure 5 and Supplemental Figure S1. On day 3, the wheat bran treatment significantly increased the *Pediococcus* abundance but decreased the *Lactococcus* and *Pectobacterium* abundances compared with Con. W1 and W2 exhibited a higher (*p* < 0.05) *Lactobacillus* abundance but lower (*p* < 0.05) *Leuconostoc* and *Pantoea* abundances than Con, while W3 exhibited a higher (*p* < 0.05) *Pantoea* abundance than Con. In addition, a higher (*p* < 0.05) abundance of *Enterobacter* was observed in W1 and W3, and a higher (*p* < 0.05) abundance of *Weissella* was observed in W3 than in Con. In contrast, the rice bran treatment significantly increased the *Pediococcus* abundance but decreased the *Lactococcus* and *Pantoea* abundances compared with Con. Furthermore, R1 and R2 exhibited higher (*p* < 0.05) *Enterobacter* and *Pectobacterium* abundances than Con, while R3 exhibited a lower (*p* < 0.05) *Pectobacterium* abundance than Con. Moreover, R1 and R3 exhibited higher (*p* < 0.05) *Lactobacillus* abundances than Con.

On day 60, the wheat bran treatment significantly increased the *Pediococcus* and *Enterobacter* abundances but decreased the *Lactococcus* abundance compared with Con. W2 and W3 significantly increased the *Weissella* abundance compared with Con, while W1 exhibited a lower (*p* < 0.05) *Leuconostoc* abundance than Con. The rice bran treatment significantly decreased the *Lactococcus* and *Leuconostoc* abundances compared with Con, while R3 possessed a higher (*p* < 0.05) *Enterobacter* abundance than Con.

The Lefse analysis was employed to reveal the biomarker of the microbial community. On day 3, *Enterobacter* and *Lactobacillus* exhibited an LDA score exceeding 4.0 in W3 and W2, respectively. High LDA scores of *Enterobacter* and *Pectobacterium* were observed in R2 and R3, respectively. On day 60, *Pediococcus*, *Weissella* and *Enterobacter* exhibited LDA scores exceeding 4.0 in W3, W2, and W1, respectively.

### 3.6. Co-occurrence network of the bacteriome

**Figure 6:**
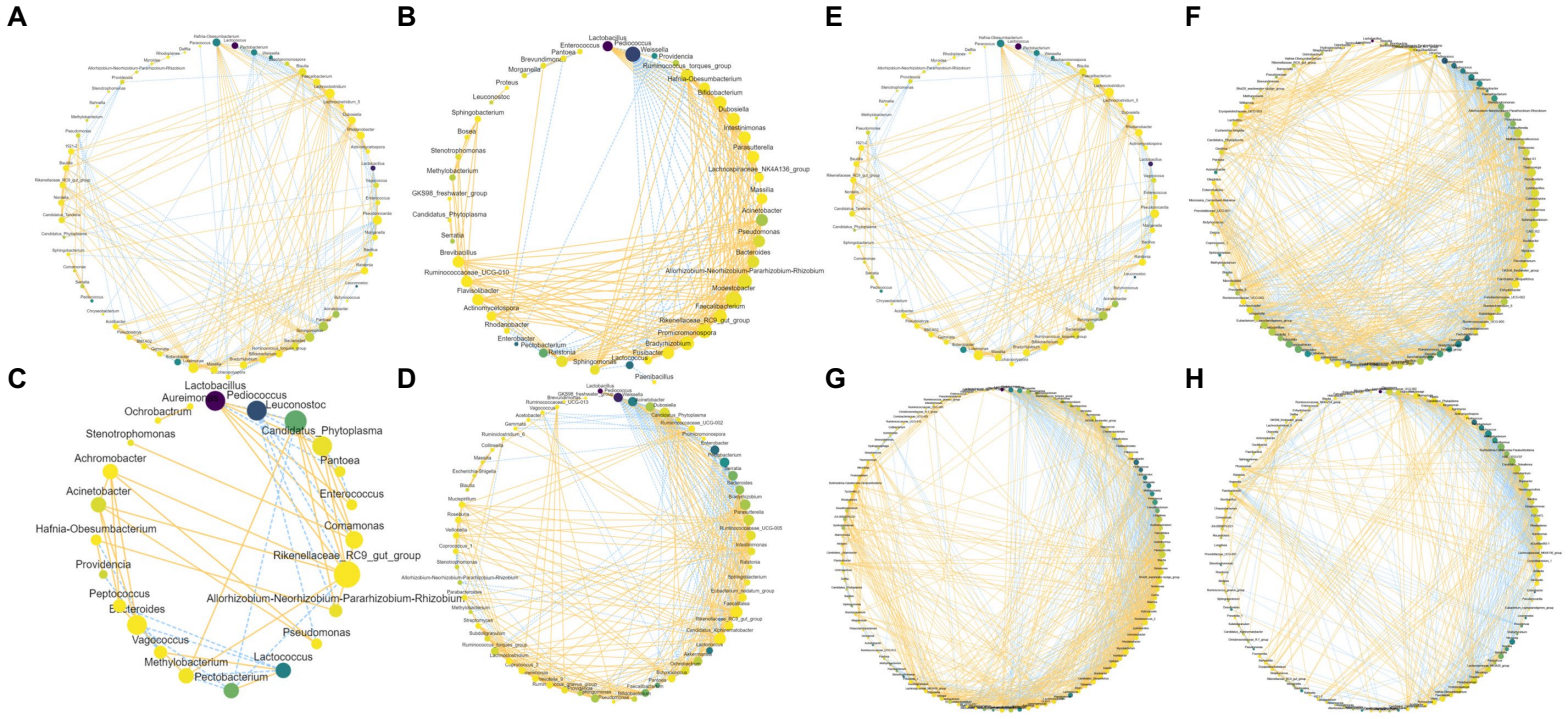
Co-occurrence network within bacteriome in Chinese cabbage waste fermented alone **(A,E)** and with wheat bran (**B**, W1; **C**, W2; **D**, W3) or with rice bran (**F**, R1; **G**, R2; **H**, R3). The node size is proportional to the connectivity of the node. The edge color represents the negative (blue) correlation and positive (yellow) correlation.

Co-occurrence networks of the bacteriome are shown in Figures 6A–D. W1 and W2 decreased the counts of node and edge compared with Con, whereas W3 exhibited higher counts of node and edge than Con. Moreover, the wheat bran treatment increased the positive edge ratios compared with Con. As shown in Figures 6E–H, the rice bran treatment groups exhibited higher counts of node and edge than Con, and R2 possessed the highest values. In addition, R2 resulted in a higher positive edge ratio than that of Con.

### 3.7. Correlation between environmental factors and bacteria

**Figure 7:**
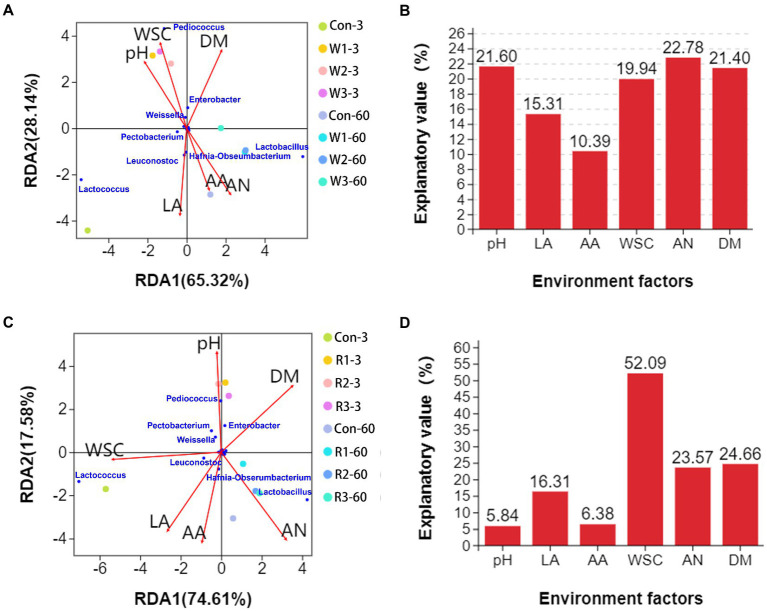
RDA of environmental factors, samples and bacterial genera in Chinese cabbage waste anaerobically fermented with wheat bran **(A)** or with rice bran **(C)**. VPA of the microflora explained by the environmental factors in Chinese cabbage waste anaerobically fermented with wheat bran **(B)** or rice bran **(D)**. The control group (Con). Chinese cabbage waste was mixed with wheat bran at a mass ratio of 383:117 (W1), 353:147 (W2), and 323:177 (W3) or with rice bran at 387:113 (R1), 358:142 (R2), and 329:171 (R3), respectively; 3 and 60 mean day 3 and day 60, respectively; DM, dry matter; LA, lactic acid; AA, acetic acid; AN, ammonia-N; WSC, water-soluble carbohydrate.

Among the wheat bran treatment groups, RDA (Figure 7A) analysis indicated that *Lactobacillus*, *Leuconostoc* and *Hafnia*-*Obseumbacterium* abundances positively correlated with the contents of lactic acid, acetic acid, and ammonia-N but negatively correlated with the pH value and content of WSC. *Lactobacillus* abundance positively correlated with the DM content, whereas *Leuconostoc* and *Hafnia*-*Obseumbacterium* abundances negatively correlated with the DM content. *Pediococcus*, *Weissella* and *Enterobacter* abundances positively correlated with the pH value and contents of WSC and DM but negatively correlated with the contents of lactic acid, acetic acid and ammonia-N. In addition, *Lactococcus* abundance had positive correlations with pH value and contents of lactic acid, acetic acid and ammonia-N but possessed negative correlations with the contents of WSC and DM. VPA analysis (Figure 7B) shows that environmental factors, including pH, WSC, ammonia-N and DM, exhibited relatively high explanatory values, while ammonia-N exhibited the highest value.

Among the rice bran treatment groups, RDA (Figure 7C) analysis indicated that *Lactococcus*, *Leuconostoc* and *Hafnia*-*Obseumbacterium* abundances positively correlated with the contents of lactic acid, acetic acid and WSC but negatively correlated with the pH value and DM content. In addition, *Lactococcus* and *Leuconostoc* abundances had negative correlations with the ammonia-N content. *Pediococcus* and *Enterobacter* abundances positively correlated with the pH value and DM content but negatively correlated with the contents of lactic acid, acetic acid, WSC and ammonia-N. *Weissella* and *Pectobacterium* abundances had positive correlations with the pH value and contents of WSC and DM but possessed negative correlations with the contents of lactic acid, acetic acid and ammonia-N. *Lactobacillus* abundance positively correlated with the contents of lactic acid, acetic acid, ammonia-N and DM but negatively correlated with the pH value and WSC content. As shown in Figure 7D, WSC possessed the highest explanatory value for variations in bacteriome composition when introducing rice bran.

### 3.8. Predicted functional profiles and phenotypes of bacterial communities

**Figure 8:**
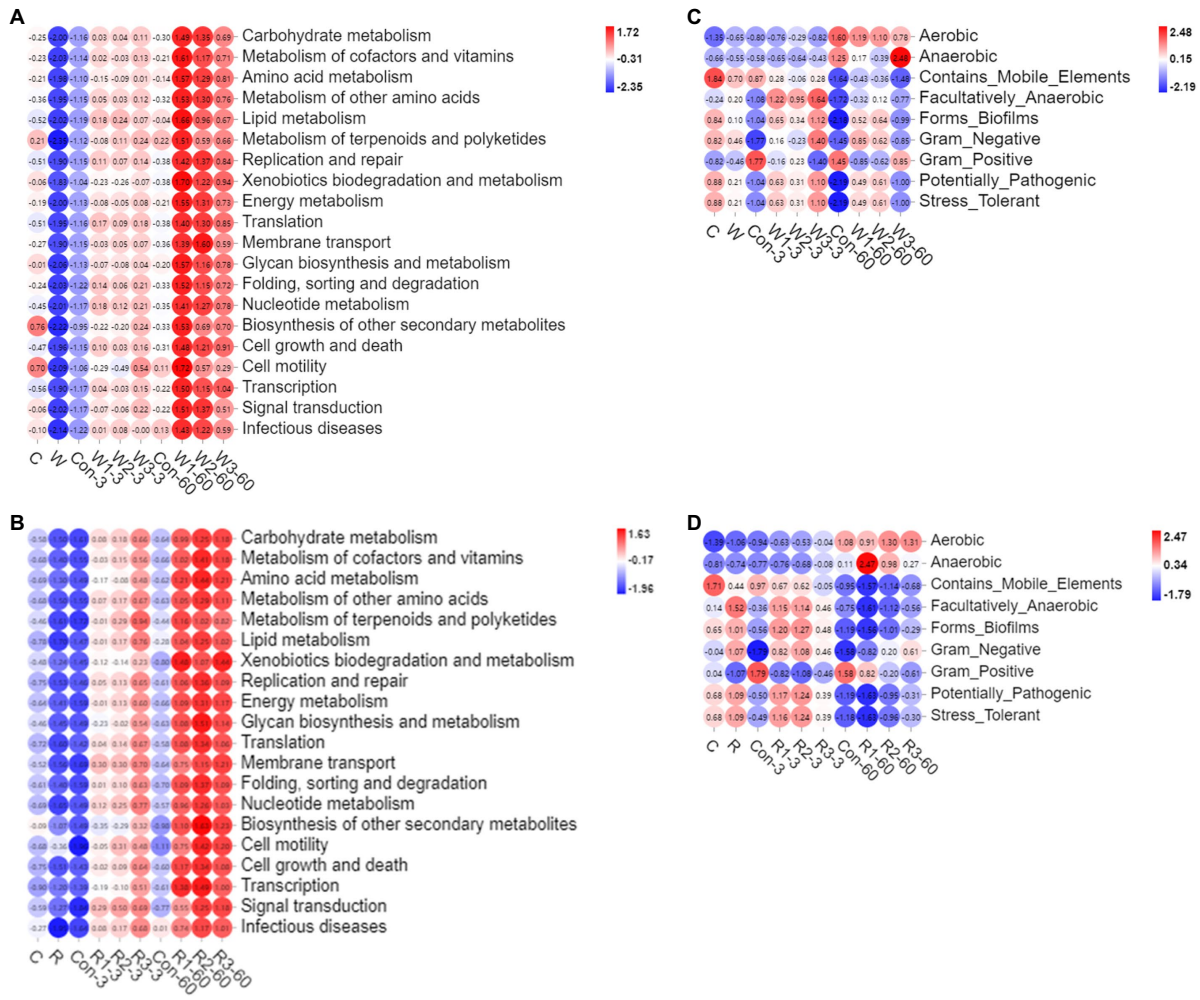
16 rRNA-predicted functional profiles were obtained with PICRUSt2 in Chinese cabbage waste anaerobically fermented with wheat bran **(A)** or rice bran **(B)**. The bacterial community phenotypes were obtained with BugBase in Chinese cabbage waste anaerobically fermented with wheat bran **(C)** or rice bran **(D)**. The control group (Con). Chinese cabbage waste was mixed with wheat bran at a mass ratio of 383:117 (W1), 353:147 (W2), and 323:177 (W3) or with rice bran at 387:113 (R1), 358:142 (R2), and 329:171 (R3), respectively; 3 and 60 mean day 3 and day 60, respectively; raw Chinese cabbage waste (C), raw rice bran (R), raw wheat bran (W).

As shown in Figures 8A,B and Supplemental Figure S2, the bran treatments significantly upregulated the global metabolic pathways examined in the present study compared with Con. In addition, R3 significantly upregulated the global metabolic pathways compared with R1 on day 3, while W3 significantly downregulated the carbohydrate, cofactor, vitamin metabolism, other amino acid and energy metabolisms compared with W1 on day 60.

As shown in Figures 8C,D and Supplemental Figure S3, the bran treatments significantly increased the bacterial communities that were facultatively anaerobic, biofilm-formed, Gram-negative, potentially pathogenic and stress-tolerant but decreased the bacterial flora that was Gram-positive compared with Con on day 3. On day 60, W1 and W2 significantly increased the bacterial communities that were biofilm-formed, potentially pathogenic and stress-tolerant compared with Con, while R3 significantly increased the Gram-negative bacteria but decreased Gram-positive ones compared with Con.

## 4. Discussion

The high moisture level that benefits effluent formation suggests that introducing a moisture adjuster can avoid nutrient loss and hygienic problems during the Chinese cabbage waste fermentation process. Okine et al. (2007) reported that the brans showed poorer effluent retention capacities than bean stalks and husks, straws and dried beet pulp. However, the bran treatments in the present study undoubtedly eliminated effluent formation. In addition, wheat bran and rice bran, with essential nutrients and bioactive substances, are suitable for transforming Chinese cabbage waste into livestock feed (Rosenfelder et al., 2013; Sharif et al., 2014).

A pH value of 3.8–4.2 could inhibit most of the undesired microorganisms, and an excessively low pH might impair palatability (Ren H. et al., 2019). In the present study, except for Con and R3, the other groups exhibited potential as livestock feed. A previous study indicated that an increase in DM contents limits LAB metabolisms, which attenuates the organic acid production, followed by a slow decline rate of pH value (Kung et al., 2018). In addition, among organic acids, lactic acid is regarded as the most effective acid contributing to the decline of pH value due to the low pKa value (Kung et al., 2018). Thus, the increase in DM contents caused by introducing the brans might be a potential reason for the low lactic acid levels and high pH values in the bran treatment groups and the groups with a high DM level. However, the lack of difference in pH value between Con and W1 at the early fermentation stage indicated that alkaline substances, such as ammonia-N, were massively produced in Con when the fermentation was initiated, which was inhibited in the wheat bran treatment groups. In addition, the antagonism between indigenous and exogenous microorganisms restricts organic acid fermentation, suggesting that more time is necessary to reestablish a stable microbiome when the brans are introduced (Dong Z. H. et al., 2020). This could also explain the phenomenon that the bran treatment groups with high DM contents possessed a similar lactic acid concentration and pH value to those with low DM contents at the final stage. Short-chain fatty acids are crucial for anaerobic fermentation. Furthermore, short-chain fatty acids can be antimicrobial molecules to inhibit the growth of microorganisms and maintain the stability of the fermentative system (Li J. et al., 2022). Surprisingly, the lack of reduction in acetic acid levels was observed between Con and bran treatment groups, except for R2, at the final stage of fermentation. The acetic acid production in anaerobic fermentation mainly results from heterofermentation, which utilizes one mole of glucose to produce one mole of lactic acid, one mole of acetic acid and one mole of carbon dioxide. A previous study indicated that the high DM content suppressed the metabolism of *Lactobacillus buchneri*, which is an obligate heterofermentative LAB, followed by a reduction in acetic acid production (Restelatto et al., 2019). This could explain the reduction in acetic acid concentration in the bran treatment groups at the early fermentation stage. However, with the decrease in pH value, the change in transmembrane pH value gradient shifts the products with a low pKa value to the products with a pKa value, such as acetic acid, which could explain the increase in acetic acid in the bran treatment groups at the final stage, wherein had lower (*p* < 0.05) pH values than at early stage (Oude Elferink et al., 2001; Kung et al., 2018). Moreover, despite the low organic acid levels, the enhanced WSC consumption in the wheat bran treatment groups on day 1 suggested that introducing wheat bran might stimulate the growth of undesired microbes. On the other hand, the porous structure of wheat bran was easy to cause extra residual oxygen, which supports the growth of aerobic bacteria at the early stage, resulting in excess WSC consumption (De Bondt et al., 2020). In addition, the insignificant difference in WSC was observed among wheat bran treatment groups and rice bran treatment groups, respectively, indicating that microbial metabolism might be the main reason for the variation in WSC content, whereas the rapid reduction in WSC in Con was partly attributed to the massive effluent formation (Yang et al., 2019). Moreover, Wang et al. (2017) demonstrated that the epiphytic microbiome in the different materials possessed different conversion efficiency of substrates to lactic acid, which probably explained the high WSC contents in the wheat bran treatment groups from days 3–60 due to the high conversion efficiency of the reestablished microbiome. In addition, an increase in DM content, which contributed to inhibiting the growth of undesired microbes, was also a potential reason for enhanced WSC preservation (He et al., 2020c). In contrast, the rice bran treatment groups exhibited a lower (*p* < 0.05) WSC content than the wheat bran treatment groups due to the difference in WSC contents between fresh wheat bran and rice bran, indicating that the insufficient fermentative substrate might be another main reason for the weak fermentation intensities and low WSC content in rice bran treatment groups. Furthermore, despite the increase in DM levels in R3 compared with R1 and R2, the reduction in WSC level indicated that introducing rice bran might promote the growth or metabolism of undesired microorganisms, such as *Enterobacter* competed for available substrate with LAB (He et al., 2020c). Interestingly, no propionic acid generation was observed in Con, R1, R2, and R3, but the propionic acid was detected in the wheat bran treatment groups, suggesting the presence of certain microbes, such as *Lactobacillus diolivorans*, which degrades 1,2-propanediol, a product of obligate fermentative *Lactobacillus buchneri*, to propionic acid and 1-propanol (Krooneman et al., 2002).

The culturable microbial profile is a key parameter in evaluating the fermentation efficiency of a fermentation system. Although the bran treatments weakened fermentation intensity, the bran treatments possessed high LAB counts. This contradictory phenomenon might be caused by the low pH value in Con, which could also inhibit the growth of LAB, especially for the acid-sensitive LAB, such as *Pediococcus*, which is an early colonizer in the anaerobic fermentation and possesses a rapid growth rate when the pH ranges from 5 to 6.5 (Wang et al., 2019). Dong Z. H. et al. (2020) demonstrated that the interaction between the epiphytic microbiome from different materials extended the time desired to stabilize the microbiome succession. Therefore, the results in the present study indicated that the LAB consortia of W1 were difficult to become stable, and more time was necessary. Coliform bacteria consist of *Pseudomonas*, *Enterobacteriaceae* and *Aeromonas*, which are always associated with nutrient loss and the failure of fermentation (Wang et al., 2020; He et al., 2020e). He et al. (2020c) reported that the increase in DM contents limits the growth of coliform bacteria, which was consistent with the results of the bran treatment groups in the present study. Notably, the low pH value inhibits the growth of coliform bacteria, which could explain the increase in coliform bacteria counts in W3, R1 and R2 from days 5–15 (Li J. et al., 2022). These results also indicated that the DM content and pH value might jointly influence the coliform bacteria counts. Furthermore, yeast utilizes lactic acid to produce ethanol and carbon dioxide in anaerobic environments, causing DM loss and spoilage (de Jesus et al., 2019). In general, acetic acid is the main antifungal molecule, which can penetrate the cell membrane in an undissociated form and release protons to inhibit fungi growth (Ren H. W. et al., 2019). In the present study, despite the low acetic acid levels observed in the bran treatment groups from days 1–30, the bran treatments also decreased the yeast counts. Notably, the pH value influences the antimicrobial activity of organic acids, such as the high pH value can cause acetic acid or propionic acid to exist more in an undissociated form (Skrivanova and Marounek, 2007). In addition, organic acids synergistically exert bactericidal capacity (Burns et al., 2021). Thus, the bran treatments benefited in constructing an environment that inhibited the growth of undesired microorganisms.

The increase in the crude protein level was associated with the formation of gas and effluent during the anaerobic fermentation, which was consistent with the present results that enhanced heterofermentation was observed in all the groups at the final stage (Li et al., 2019). In addition, the protein degradation process during the anaerobic fermentation was divided into two stages, as follows: (1) the proteases from the plant hydrolyze the peptide linkage of true protein to produce the metabolic intermediates, such as peptides and free amino acids; (2) the intermediates were further degraded to ammonia or other final products, which was driven by microbial enzymes (He et al., 2019a). Therefore, the nonprotein-N and ammonia-N levels increased with the fermentation process. In addition, proteolytic enzymes from plants and microbes become active when the pH value ranges from 5 to 6 (He et al., 2020a). The bran treatments inhibited true protein degradation in the present study, probably due to the decline in harmful proteolytic bacteria. However, the wheat bran treatment promoted nonprotein-N formation on day 60. Nogata and Nagamine (2009) reported that the optimal pH value for endopeptidases in wheat bran is 4.0, indicating that the fermentation environment constructed in the wheat bran treatment groups cannot completely inhibit proteolysis. Free amino acid-N and ammonia-N contents are precise indicators for evaluating peptide linkage degradation and deamination (He et al., 2020a). The low free amino acid-N contents in the bran treatment groups indicated that introducing the brans reduced free amino acid accumulation induced by endopeptidases or oligopeptidases at the early stage. However, the bran treatment groups possessed higher free amino acid-N contents than Con at the final stage. During the anaerobic fermentation process, microbes produce alkaline substances to resist the acid threat (Kieliszek et al., 2021). Thus, the high pH value induced by the bran treatments might reduce ammonia-N formation, which resulted in free amino acid accumulation by reducing the deamination at the final stage. In addition, the reduction in undesired microorganisms caused by the bran treatments, such as coliform bacteria and yeast, was conducive to decreasing ammonia-N content. Ammonia-N formed by deamination is a troubling pollutant that could be reacted with sulfuric and nitric acids to form fine particles, causing air pollution on the farm. In addition, the ammonia nitrogen emission threatens animal and human health and could be transported over long distances in the atmosphere (Gu et al., 2020). Therefore, introducing the brans was conducive to inhiting the alkaline pollutant generaction, which might reduce the risk of air pollution. Moreover, an ammonia-N level below 100 g/kg TN is recommended, which indicates little protein degradation and that the protein quality of the fermentation product is acceptable (Ren H.W. et al., 2019). Herein, Con and the rice bran treatment groups exhibited high ammonia-N levels, and the wheat bran treatment groups are more suitable fermentation systems for recycling Chinese cabbage waste as livestock feed.

Dietary antioxidant supplementation could improve the antioxidative status and meat quality of the animal, indicating that evaluating the antioxidant activity is crucial for estimating the quality of fermentative products (Meng et al., 2020; Li J. B. et al., 2022). He et al. (2020b) reported that a high organic acid concentration could disrupt the bioactive components, reducing antioxidant activity. Therefore, although the wheat bran treatment resulted in a weak fermentation intensity, high antioxidant activities were observed in the wheat bran groups. In addition, bioactive peptides and metabolites of LAB contributed to increasing antioxidant activity, as reflected by the high free amino acid level and LAB counts in the wheat bran treatment groups (Li et al., 2021). However, the rice bran treatment groups exhibited low antioxidant activities. Combined with the DPPH, ABTS, and FRAP assays results, we proposed that the rice bran treatment reduced antioxidant activity by decreasing the radical scavenging that involves the hydrogen-atom transfer and decreasing single electron transfer. Furthermore, although it improved antioxidant activities, the wheat bran treatment groups possessed DPPH levels of 2.06–4.31 mg of trolox equivalent/g DM, ABTS levels of 8.43–8.72 trolox equivalent/g DM, FRAP levels of 3.16–3.81 trolox equivalent/g DM, which was far lower than the levels in *Moringa oleifera* leaves, indicating that the antioxidant activities of the fermentation products based on Chinese cabbage waste and wheat bran might few cause the positive effects on animals (He et al., 2020b). Fiber influences the development and morphology of tract and microbiome structure, while a high fiber level reduces the digestive efficiency of feed (Shi et al., 2021). In the present study, the rice bran treatment increased the NDF and ADF contents, while the wheat bran treatment increased the NDF contents but decreased the ADF contents. Cellulose, hemicellulose and lignin are the major components in NDF, while ADF mainly contains cellulose and lignin, which are invalid fiber fractions for nutritional use by animals (Van Soest et al., 1991). Therefore, introducing rice bran might reduce digestibility, but introducing wheat bran might stimulate microbes in the intestinal tract to produce short-chain fatty acids by increasing hemicellulose.

Venn diagrams, Upset plots, and PCoA reflected the variations in the microbiome (Zhao et al., 2022). The discernible separation in PCoA indicated that the bran treatments changed the bacteriome structure. However, a similar bacteriome structure was observed among the bran treatment groups. Dong L. et al. (2020) found that fermenting *Broussonetia papyrifera* with perennial at different ratios had a similar bacteriome structure, which was consistent with the present study. Therefore, a hypothesis was proposed that redundant microbes existed among the bran treatment groups. Anaerobic and acidic stresses resulted in the limitation of the growth of undesired microorganisms, reducing the core OTU counts, as shown in W3 but inconsistent with the other groups (Du G. L. et al., 2021). This contradiction was probably attributed to the raw materials enriched with LAB and WSC, which contributed to rapidly constructing anaerobic and acidic environments that stimulated the growth of LABs.

*Pediococcus* vigorously grows under neutral conditions, and the excessively acidic circumstance inhibits its metabolism (Dong Z. H. et al., 2020). In addition, a condition with a high DM content is conducive to *Lactobacillus* in anaerobic fermentation (Li F. H. et al., 2020). Thus, the enriched *Pediococcus* and *Lactobacillus* in the bran treatment groups might result from the constructed environment, which had a high pH value and a high DM content. In addition, the growth of *Pediococcus* could stimulate the enrichment of *Lactobacillus*, which contributed to the reduction in protein degradation and the stability of the fermentation system, as shown in the present study (Dong Z. H. et al., 2020). Moreover, *Lactococcus* and *Leuconostoc* are always found under the condition with a high pH value because they are sensitive to acidic conditions (Dong Z. H. et al., 2020). However, the present study observed the opposite phenomenon, which was hard to explain. Keshri et al. (2019) indicated that the metabolites from certain bacterial strains could be utilized by other bacteria, which results in a synergism, causing variations in the microbiome structure. Therefore, interactions between microbes should be investigated to reveal the underlying mechanisms further. Moreover, *Lactococcus* is a homofermentative LAB, and lactic acid is the only fermentation end product (Nazar et al., 2020). *Leuconstoc* and *Weissella* are heterofermentative LABs, fermenting WSC to produce lactic acid, acetic acid and carbon dioxide as the fermentation end products (Dong Z. H. et al., 2020; Nazar et al., 2020). Therefore, the reduction in *Leuconstoc* and *Lactococcus* abundances might be the main reason for the reduction in lactic acid and acetic acid levels in the bran treatment groups, and the enrichment of *Weissella* might be the reason for the high level of acetic acid in the wheat bran treatment groups at the final stage. Moreover, *Pantoea* is regarded as an indicator of food spoilage, which belongs to the *Enterobacteriaceae* family (Pan et al., 2019). *Pectobacterium* has been identified as spoilage-induced microbes during carrot storage (Kahala et al., 2012). In addition, *Enterobacter*, a facultatively anaerobic microbe, can ferment lactic acid to acetic acid and other products, which causes nutrient loss and decreases fermentation quality (Ren et al., 2018). Therefore, the present results indicated that although the constructed environment induced by the bran treatments inhibited some spoilage microbes, some undesired microbes might enrich in the bran treatment groups due to the slow decline in pH value.

Node and edge counts positively correlate with the size and complexity of the microbiome, and the positive edge ratio reflects the relationship within the microbiome (Banerjee et al., 2016). In the present study, the wheat bran treatment promoted commensalism or mutualism, while W3 improved the scale and complexity of the bacteriome. In addition, the rice bran treatment increased the scale and complexity of the bacteriome, while only R2 enhanced commensalism or mutualism. Bello et al. (2020) reported that nutrient availability enhancement could induce an extensive and well-connected co-occurrence network. Banerjee et al. (2016) also found that providing extra nutrients could enhance commensalism or mutualism. However, the rice bran treatment with a low substrate content constructed an extensive and well-connected network, while W1 and W2 with a high substrate level reduced the scale and complexity. Thus, we proposed that the weak fermentative intensity caused by the bran treatments enhanced nutrient availability, resulting in increases in the scale and complexity of the networks. In addition, a large and complex co-occurrence network has a high resistance and remains stable when receiving exogenous stimuli (Lei et al., 2021). Therefore, W3 and R2 might be the more suitable fermentation systems for the resource recycling of Chinese cabbage waste due to the high-resistance bacteriome.

RDA and VPA contribute to revealing the specific roles of a certain microbe in a given ecosystem. In the present study, introducing wheat bran unquestionably improved the DM content of the fermentative system, which enriched with *Pediococcus*, *Weissella*, *Lactobacillus* and *Enterobacter* but inhibited *Lactococcus*. The positive correlation between *Lactococcus* and organic acids indicated that the reduction in *Lactococcus* abundance was the main reason for the weak fermentation intensity. In addition, ammonia-N exhibited a high explanatory value for the variations in the bacteriome structure when introducing wheat bran. Ammonia-N could be utilized by microbes as a nitrogen source (Kieliszek et al., 2021). Pedersen et al. (2013) reported that the growth of *Leuconostoc*, which has low aminopeptidase activity, benefited from the proteolytic activity of *Lactococcus*. Therefore, the low ammonia-N content resulted in a reduction in *Leuconostoc* abundance when introducing wheat bran, which further weakened fermentative intensity. The rice bran treatment also increased *Pedicococcus*, *Enterobacter* and *Lactobacillus* abundances but decreased *Lactococcus* and *Leuconostoc* abundances. However, on day 60, introducing rice bran only increased *Enterobacter* abundance. As shown in VPA, WSC showed the highest explanatory value for the differences in bacteriome structure when the rice bran was introduced. Therefore, we speculated that improving DM content stimulated *Pediococcus*, *Enterobacter* and *Lactobacillus* but inhibited *Lactococcus* and *Leuconostoc* when rice bran was introduced, which resulted in the weak fermentative intensity and low ammonia-N content. The insufficient fermentative substrate might be why there are no differences in *Pediococcus* and *Lactobacillus* abundances between Con and rice bran treatment groups on day 60, which resulted in relatively high ammonia-N contents.

Microbes degrade substrates and synthesize metabolites by metabolic pathways during anaerobic fermentation. Predicting bacterial function provides keystone information for assessing fermentation performance (Du et al., 2021b). Du G. L. et al. (2021) reported that a high moisture level is conducive to upregulating the global metabolic pathways, while pressures from adverse environments, such as acid and anaerobic conditions, repress the metabolism of aerobic microorganisms. Although introducing the brans increased DM contents in the present study, the bran treatments upregulated the global metabolic pathways, which was inconsistent with the above study. Bai et al. (2022) indicated that a high total LAB abundance leads to upregulating the carbohydrate and nucleotide metabolism pathways. Therefore, the upregulation of the global metabolic metabolism probably resulted from the LAB proliferation stimulated by the constructed environment with a high pH value and DM level. Furthermore, carbohydrate metabolism, including pyruvate, glycolysis and butanoate metabolisms, reflects not only WSC consumption but also taste and palatability (Du et al., 2021b). Amino acid metabolism reflects the capacity to degrade the high-molecular-weight protein into amino acids and peptides, including ammonia formation and functional substrate production, such as antimicrobial and bioactive peptides (Li J. et al., 2020; Lai et al., 2022; Wang et al., 2022; Dou et al., 2023). Therefore, the high incidence of the global metabolism pathways in the bran treatment groups is conducive to improving the quality of the final fermentative products. The above results also suggested that investigating metabolome is necessary to precisely assess the nutrient value of fermentative products from Chinese cabbage waste.

Phenotypic characteristics of the microbial community are tightly correlated with ecological function characteristics. In the present study, microorganisms that were aerobic, facultatively anaerobic, biofilm-formed, Gram-negative, potentially pathogenic, and stress-tolerant increased in the bran treatment groups on day 3. Du et al. (2021a) indicated that exogenous probiotic inoculation and the anaerobic environment decreased the bacterial communities that were Gram-negative, potentially pathogenetic, mobile element-contained, and biofilm-formed. Cao et al. (2021) indicated that destroying anaerobic environments increased microorganisms that were aerobic, facultatively anaerobic, mobile element-contained, biofilm-formed, and potentially pathogenic phenotypes and a reduction in those that were anaerobic phenotypes. Therefore, the effects of the bran treatments on day 3 on the phenotypic characteristics of the microbial community were adverse, which probably resulted from that introducing the brans increased residual oxygen in the fermentative system. Moreover, establishing a new balance under anaerobic conditions resulted in the reduction in bacteria that were mobile element-contained, biofilm-formed, and potentially pathogenic, which was consistent with the phenomenon of the present study on day 60 (Cao et al., 2021). Furthermore, a high incidence of aerobic bacteria raises nutrient loss and leads to aerobic spoilage. Bacteria that are biofilm-formed can induce various infections and cause risks to animal health (Shang et al., 2021). Therefore, the mixed ratios in W3 and R2 were recommended for constructing a system to recycle Chinese cabbage waste from a microbiological safety viewpoint.

The combination results of the bacteriome indicated that the wheat bran treatment enriched *Pediococcus* and *Weissella*, which contributed to the reduction in ammonia-N content and the improvement in WSC level and acetic acid production, whereas reduced *Lactococcus*, which was positively associated with the pH decline and lactic acid production. In contrast, the rice bran treatment groups enriched *Lactobacillus* and *Pediococcus* but reduced the *Lactococcus* abundance, which contributed to the increase in pH value and the reduction in organic acid production. Furthermore, the bran treatments upregulated the global metabolic pathways, which was associated with the LAB growth, nutritive value and palatability. Finally, the results also indicated that the bran treatments might increase the risk of microbial safety.

The limitations and prospects of the present study were also proposed as follows: (1) the enhanced WSC preservation was easy to induce aerobic spoilage, which should be determined and avoided in future studies; (2) although it decreased the ammonia-N formation, the wheat bran treatment enhanced true protein degradation, indicating that an inhibitor against proteolysis should be introduced; (3) the relatively high pH value in wheat bran treatment groups indicated that introducing an acidifier might improve the fermentation quality; (4) the lack of WSC in rice bran caused the negative effects on the fermentation system based on Chinese cabbage waste and rice bran, indicating that introducing carbon sources might a suitable method. Moreover, although it weakened the fermentation intensity, W3 exhibited an acceptable pH value, the highest WSC preservation and the lowest nonprotein-N and ammonia-N levels among the wheat bran treatment groups, indicating that nutrient losses were few formed and the fermentation products of W3 might be suitable for as livestock feed (Li et al., 2023b). In addition, the lower (*p* < 0.05) abundance of undesired microbes (potentially pathogenic, stress-tolerant, and biofilm-formed) in W3 than that in W1 and W2 also indicated that the microbial safety of the fermentation products of W3 was better than that of W1 and W2 (Li et al., 2023a). Furthermore, among the rice bran treatment groups, R3 showed an unacceptable pH value after 60 days of fermentation, thereby R1 and R2 were more suitable (Ren H. et al., 2019). Moreover, R2 showed lower (*p* < 0.05) nonprotein-N and ammonia-N levels than R1, indicating that the fermentation system of R2 was recommended from the nutrient loss viewpoint (Li et al., 2023b). Therefore, the present study only provided a basic platform for recycling Chinese cabbage waste into livestock feed, and further studies on improving fermentation quality were desired.

The present study revealed that the bran treatments improved LAB population and protein preservation and reduced effluent formation, organic acid production, undesired microorganisms and alkaline pollutant generation during Chinese cabbage waste fermentation. In addition, the wheat bran treatment resulted in a low fermentative substrate consumption but enhanced true protein degradation and antioxidant activity. Microbiologically, W3 and R2 changed dominated bacteria, improved the resistance of bacteriome, and upregulated global metabolism pathways but caused adverse effects on phenotypic characteristics at the early stage of fermentation. Collectively, the mixed ratios in W3 and R2 are suitable for constructing fermentation systems for transforming Chinese cabbage waste into livestock feed from fermentation quality, nutrient preservation, pollutant generation and microbiological safety viewpoints.

## Data availability statement

The datasets presented in this study can be found in online repositories. The names of the repository/repositories and accession number(s) can be found at: https://www.ncbi.nlm.nih.gov/, PRJNA911242.

## Author contributions

JiawL: conceptualization, methodology, data curation, visualization, and writing—original draft. CW: resource, methodology, and investigation. SZ: visualization, formal analysis, and methodology. JX: resource, formal analysis, and visualization. CS: methodology and investigation. QM: methodology and formal analysis. JianL: investigation. SJ: supervision and methodology. AS: conceptualization, methodology, and supervision. All authors contributed to the article and approved the submitted version.

## Funding

This work was supported by the National Key R&D Program of China (no. 2022YFD1300600), the National Natural Science Foundation of China (nos. 32030101 and 32272914), and the Heilongjiang Touyan Innovation Team Program, and the China Agriculture Research System of MOF and MARA.

## Conflict of interest

The authors declare that the research was conducted in the absence of any commercial or financial relationships that could be construed as a potential conflict of interest.

## Publisher’s note

All claims expressed in this article are solely those of the authors and do not necessarily represent those of their affiliated organizations, or those of the publisher, the editors and the reviewers. Any product that may be evaluated in this article, or claim that may be made by its manufacturer, is not guaranteed or endorsed by the publisher.

## References

[ref1] AOAC (1990). Official methods of analysis. 15th Edn. Association of Official Analytical Chemists. Arlington, VA: AOAC.

[ref2] Bai J. Franco M. Ding Z. Hao L. Ke W. Wang M.. (2022). Effect of *Bacillus amyloliquefaciens* and *Bacillus subtilis* on fermentation, dynamics of bacterial community and their functional shifts of whole-plant corn silage. J. An. Sci. Biotechnol. 13:7. doi: 10.1186/s40104-021-00649-0, PMID: 34991716 PMC8739699

[ref3] Banerjee S. Kirkby C. A. Schmutter D. Bissett A. Kirkegaard J. A. Richardson A. E. (2016). Network analysis reveals functional redundancy and keystone taxa amongst bacterial and fungal communities during organic matter decomposition in an arable soil. Soil Biol. Biochem. 97, 188–198. doi: 10.1016/j.soilbio.2016.03.017

[ref4] Bello A. Han Y. Zhu H. F. Deng L. T. Yang W. Meng Q. X.. (2020). Microbial community composition, co-occurrence network pattern and nitrogen transformation genera response to biochar addition in cattle manure-maize straw composting. Sci. Total Environ. 721:137759. doi: 10.1016/j.scitotenv.2020.137759, PMID: 32172117

[ref5] Broderick G. A. Kang J. H. (1980). Automated simultaneous determination of ammonia and Total amino acids in ruminal fluid and in vitro media. J. Dairy Sci. 63, 64–75. doi: 10.3168/jds.S0022-0302(80)82888-8, PMID: 7372898

[ref6] Burns J. McCoy C. P. Irwin N. J. (2021). Synergistic activity of weak organic acids against uropathogens. J. Hosp. Infect. 111, 78–88. doi: 10.1016/j.jhin.2021.01.024, PMID: 33545217

[ref7] Cao Y. R. Zhu B. Z. Li F. Zhang D. H. Guo T. Q. Li F. D.. (2021). The destruction of the anaerobic environment caused by rumen fistula surgery leads to differences in the rumen microbial diversity and function of sheep. Front. Vet. Sci. 8:754195. doi: 10.3389/fvets.2021.754195, PMID: 34926637 PMC8671607

[ref8] De Bondt Y. Liberloo I. Roye C. Windhab E. J. Lamothe L. King R.. (2020). The effect of wet milling and cryogenic milling on the structure and physicochemical properties of wheat bran. Foods 9. doi: 10.3390/foods9121755, PMID: 33260871 PMC7759771

[ref9] de Jesus D. L. S. Rigueira J. P. S. Moncao F. P. Alves W. S. Moura M. M. A. de Sales E. C. J.. (2019). Nutritive value of sugarcane silages added with increasing levels of acetic acid. Semina-Ciencias Agrarias 40, 2387–2396. doi: 10.5433/1679-0359.2019v40n5Supl1p2387

[ref10] Dong Z. H. Shao T. Li J. F. Yang L. L. Yuan X. J. (2020). Effect of alfalfa microbiota on fermentation quality and bacterial community succession in fresh or sterile Napier grass silages. J. Dairy Sci. 103, 4288–4301. doi: 10.3168/jds.2019-16961, PMID: 32173011

[ref11] Dong L. Zhang H. Gao Y. Diao Q. (2020). Dynamic profiles of fermentation characteristics and bacterial community composition of Broussonetia papyrifera ensiled with perennial ryegrass. Bioresour. Technol. 310:123396. doi: 10.1016/j.biortech.2020.123396, PMID: 32388351

[ref12] Dou X. Yan D. Liu S. Gao N. Ma Z. Shi Z.. (2023). Host defense peptides in nutrition and diseases: a contributor of immunology modulation. J. Agric. Food Chem. 7, 3125–3140. doi: 10.1021/acs.jafc.2c08522, PMID: 36753427

[ref13] Du Z. Lin Y. Sun L. Yang F. Cai Y. (2021a). Microbial community structure, co-occurrence network and fermentation characteristics of woody plant silage. J. Sci. Food Agric. 102, 1193–1204. doi: 10.1002/jsfa.11457, PMID: 34343355

[ref14] Du G. L. Shi J. P. Zhang J. X. Ma Z. G. Liu X. C. Yuan C. Y.. (2021). Exogenous probiotics improve fermentation quality, microflora phenotypes, and trophic modes of fermented vegetable waste for animal feed. Microorganisms 9:18. doi: 10.3390/microorganisms9030644, PMID: 33808890 PMC8003719

[ref15] Du Z. Sun L. Chen C. Lin J. Yang F. Cai Y. (2021b). Exploring microbial community structure and metabolic gene clusters during silage fermentation of paper mulberry, a high-protein woody plant. Anim. Feed Sci. Technol. 275:114766. doi: 10.1016/j.anifeedsci.2020.114766

[ref16] Fausto-Castro L. Rivas-Garcia P. Gomez-Nafte J. A. Rico-Martinez R. Rico-Ramirez V. Gomez-Gonzalez R.. (2020). Selection of food waste with low moisture and high protein content from Mexican restaurants as a supplement to swine feed. J. Clean. Prod. 256:120137. doi: 10.1016/j.jclepro.2020.120137

[ref17] Goncales M. Nunhes T. V. Barbosa L. de Campos F. C. de Oliveira O. J. (2018). Opportunities and challenges for the use of cleaner production to reduce water consumption in Brazilian sugar-energy plants. J. Clean. Prod. 186, 353–363. doi: 10.1016/j.jclepro.2018.03.114

[ref18] Gu B. J. Song Y. Yu C. Q. Ju X. T. (2020). Overcoming socioeconomic barriers to reduce agricultural ammonia emission in China. Environ. Sci. Pollut. Res. 27, 25813–25817. doi: 10.1007/s11356-020-09154-9, PMID: 32399883

[ref19] He L. W. Chen N. Lv H. J. Wang C. Zhou W. Chen X. Y.. (2020a). Gallic acid influencing fermentation quality, nitrogen distribution and bacterial community of high-moisture mulberry leaves and stylo silage. Bioresour. Technol. 295:122255. doi: 10.1016/j.biortech.2019.122255, PMID: 31639626

[ref20] He L. W. Lv H. J. Chen N. Wang C. Zhou W. Chen X. Y.. (2020b). Improving fermentation, protein preservation and antioxidant activity of Moringa oleifera leaves silage with gallic acid and tannin acid. Bioresour. Technol. 297:122390. doi: 10.1016/j.biortech.2019.122390, PMID: 31740244

[ref21] He L. W. Lv H. J. Xing Y. Q. Wang C. You X. W. Chen X. Y.. (2020c). The nutrients in Moringa oleifera leaf contribute to the improvement of stylo and alfalfa silage: fermentation, nutrition and bacterial community. Bioresour. Technol. 301:122733. doi: 10.1016/j.biortech.2020.122733, PMID: 31935644

[ref22] He L. W. Wang C. Xing Y. Q. Zhou W. Pian R. Q. Chen X. Y.. (2020d). Ensiling characteristics, proteolysis and bacterial community of high-moisture corn stalk and stylo silage prepared with *Bauhinia variegate* flower. Bioresour. Technol. 296:122336. doi: 10.1016/j.biortech.2019.122336, PMID: 31704603

[ref23] He L. W. Wang C. Xing Y. Q. Zhou W. Pian R. Q. Yang F. Y.. (2019a). Dynamics of proteolysis, protease activity and bacterial community of Neolamarckia cadamba leaves silage and the effects of formic acid and *Lactobacillus farciminis*. Bioresour. Technol. 294:122127. doi: 10.1016/j.biortech.2019.122127, PMID: 31525585

[ref24] He L. W. Zhou W. Wang C. Yang F. Y. Chen X. Y. Zhang Q. (2019b). Effect of cellulase and *Lactobacillus casei* on ensiling characteristics, chemical composition, antioxidant activity, and digestibility of mulberry leaf silage. J. Dairy Sci. 102, 9919–9931. doi: 10.3168/jds.2019-16468, PMID: 31447142

[ref25] He L. W. Zhou W. Xing Y. Q. Pian R. Q. Chen X. Y. Zhang Q. (2020e). Improving the quality of rice straw silage with *Moringa oleifera* leaves and propionic acid: fermentation, nutrition, aerobic stability and microbial communities. Bioresour. Technol. 299:122579. doi: 10.1016/j.biortech.2019.122579, PMID: 31855660

[ref26] Ibarruri J. Goiri I. Cebrian M. Garcia-Rodriguez A. (2021). Solid state fermentation as a tool to stabilize and improve nutritive value of fruit and vegetable discards: effect on nutritional composition, in vitro ruminal fermentation and organic matter digestibility. Animals 11. doi: 10.3390/ani11061653, PMID: 34199410 PMC8227021

[ref27] Kahala M. Blasco L. Joutsjoki V. (2012). Molecular characterization of spoilage bacteria as a means to observe the microbiological quality of carrot. J. Food Prot. 75, 523–532. doi: 10.4315/0362-028x.Jfp-11-185, PMID: 22410227

[ref28] Keshri J. Chen Y. R. Pinto R. Kroupitski Y. Weinberg Z. G. Saldinger S. S. (2019). Bacterial dynamics of wheat silage. Front. Microbiol. 10:16. doi: 10.3389/fmicb.2019.01532, PMID: 31354651 PMC6632545

[ref29] Kieliszek M. Pobiega K. Piwowarek K. Kot A. M. (2021). Characteristics of the proteolytic enzymes produced by lactic acid bacteria. Molecules 26. doi: 10.3390/molecules26071858, PMID: 33806095 PMC8037685

[ref30] Krooneman J. Faber F. Alderkamp A. C. Elferink S. J. H. W. O. Driehuis F. Cleenwerck I.. (2002). *Lactobacillus diolivorans* sp. nov., a 1,2-propanediol-degrading bacterium isolated from aerobically stable maize silage. Int. J. Syst. Evol. Microbiol. 52, 639–646. doi: 10.1099/00207713-52-2-639 11931178

[ref31] Kung L. M. Shaver R. D. Grant R. J. Schmidt R. J. (2018). Silage review: interpretation of chemical, microbial, and organoleptic components of silages. J. Dairy Sci. 101, 4020–4033. doi: 10.3168/jds.2017-13909, PMID: 29685275

[ref32] Lai Z. H. Yuan X. J. Chen H. Y. Zhu Y. H. Dong N. Shan A. S. (2022). Strategies employed in the design of antimicrobial peptides with enhanced proteolytic stability. Biotechnol. Adv. 59:107962. doi: 10.1016/j.biotechadv.2022.107962, PMID: 35452776

[ref33] Lei L. S. Gu J. Wang X. J. Song Z. L. Wang J. Yu J.. (2021). Microbial succession and molecular ecological networks response to the addition of superphosphate and phosphogypsum during swine manure composting. J. Environ. Manag. 279:111560. doi: 10.1016/j.jenvman.2020.111560, PMID: 33172706

[ref34] Li J. B. Bai Y. S. Ma K. D. Ren Z. S. Li J. P. Zhang J.. (2022). Dihydroartemisinin alleviates deoxynivalenol induced liver apoptosis and inflammation in piglets. Ecotoxicol. Environ. Saf. 241:113811. doi: 10.1016/j.ecoenv.2022.113811, PMID: 35772362

[ref35] Li P. Gou W. L. Zhang Y. Yang F. Y. You H. Bai S. Q.. (2019). Fluctuant storage temperature increased the heterogeneous distributions of pH and fermentation products in large round bale silage. Grassl. Sci. 65, 155–161. doi: 10.1111/grs.12232

[ref36] Li F. H. Ke W. C. Ding Z. T. Bai J. Zhang Y. X. Xu D. M.. (2020). Pretreatment of Pennisetum sinese silages with ferulic acid esterase-producing lactic acid bacteria and cellulase at two dry matter contents: fermentation characteristics, carbohydrates composition and enzymatic saccharification. Bioresour. Technol. 295:122261. doi: 10.1016/j.biortech.2019.122261, PMID: 31645008

[ref37] Li J. Z. Li Q. K. Gao N. Wang Z. H. Li F. Li J. P.. (2021). Exopolysaccharides produced by *Lactobacillus rhamnosus* GG alleviate hydrogen peroxide-induced intestinal oxidative damage and apoptosis through the Keap1/Nrf2 and Bax/Bcl-2 pathways *in vitro*. Food Funct. 12, 9632–9641. doi: 10.1039/d1fo00277e, PMID: 34664577

[ref38] Li J. Ma D. Tian J. Sun T. Meng Q. Li J.. (2023a). The responses of organic acid production and microbial community to different carbon source additions during the anaerobic fermentation of Chinese cabbage waste. Bioresour. Technol. 371:128624. doi: 10.1016/j.biortech.2023.128624, PMID: 36642203

[ref39] Li J. Meng Q. Wang C. Song C. Lyu Y. Li J.. (2023b). The interaction between temperature and citric acid treatment in the anaerobic fermentation of Chinese cabbage waste. J. Clean. Prod. 383:135502. doi: 10.1016/j.jclepro.2022.135502

[ref40] Li J. Meng Q. Xing J. Wang C. Song C. Ma D.. (2022). Citric acid enhances clean recycling of Chinese cabbage waste by anaerobic fermentation. J. Clean. Prod. 348:131366. doi: 10.1016/j.jclepro.2022.131366

[ref41] Li J. Shang L. Lan J. Chou S. Feng X. Shi B.. (2020). Targeted and intracellular antibacterial activity against *S. agalactiae* of the chimeric peptides based on pheromone and cell-penetrating peptides. ACS Appl. Mater. Interfaces 12, 44459–44474. doi: 10.1021/acsami.0c12226, PMID: 32924418

[ref42] Licitra G. Hernandez T. M. Van Soest P. J. (1996). Standardization of procedures for nitrogen fractionation of ruminant feeds. Anim. Feed Sci. Technol. 57, 347–358. doi: 10.1016/0377-8401(95)00837-3

[ref43] Lin C. S. K. Pfaltzgraff L. A. Herrero-Davila L. Mubofu E. B. Abderrahim S. Clark J. H.. (2013). Food waste as a valuable resource for the production of chemicals, materials and fuels. Current situation and global perspective. Energy Environ. Sci. 6, 426–464. doi: 10.1039/c2ee23440h

[ref44] Meng Q. W. Sun S. S. Bai Y. S. Luo Z. Li Z. Y. Shi B. M.. (2020). Effects of dietary resveratrol supplementation in sows on antioxidative status, myofiber characteristic and meat quality of offspring. Meat Sci. 167:108176. doi: 10.1016/j.meatsci.2020.108176, PMID: 32408234

[ref45] Murphy R. P. (1958). A method for the extraction of plant samples and the determination of total soluble carbohydrates. J. Sci. Food Agric. 9, 714–717. doi: 10.1002/jsfa.2740091104

[ref46] Nazar M. Wang S. R. Zhao J. Dong Z. H. Li J. F. Kaka N. A.. (2020). The feasibility and effects of exogenous epiphytic microbiota on the fermentation quality and microbial community dynamics of whole crop corn. Bioresour. Technol. 306:123106. doi: 10.1016/j.biortech.2020.123106, PMID: 32171175

[ref47] Newbold T. Hudson L. N. Hill S. L. L. Contu S. Lysenko I. Senior R. A.. (2015). Global effects of land use on local terrestrial biodiversity. Nature 520:45. doi: 10.1038/nature14324 25832402

[ref48] Nogata Y. Nagamine T. (2009). Production of free amino acids and γ-aminobutyric acid by autolysis reactions from wheat bran. J. Agric. Food Chem. 57, 1331–1336. doi: 10.1021/jf802420w, PMID: 19170632

[ref49] Okine A. Yimamu A. Hanada M. Izumita M. Zunong M. Okamoto M. (2007). Ensiling characteristics of daikon (Raphanus sativus) by-product and its potential as an animal feed resource. Anim. Feed Sci. Technol. 136, 248–264. doi: 10.1016/j.anifeedsci.2006.09.005

[ref50] Oude Elferink S. J. Krooneman J. Gottschal J. C. Spoelstra S. F. Faber F. Driehuis F. (2001). Anaerobic conversion of lactic acid to acetic acid and 1, 2-propanediol by Lactobacillus *buchneri*. Appl. Environ. Microbiol. 67, 125–132. doi: 10.1128/aem.67.1.125-132.2001, PMID: 11133436 PMC92530

[ref51] Pan T. Xiang H. Y. Diao T. T. Ma W. Shi C. Xu Y.. (2019). Effects of probiotics and nutrients addition on the microbial community and fermentation quality of peanut hull. Bioresour. Technol. 273, 144–152. doi: 10.1016/j.biortech.2018.10.088, PMID: 30428406

[ref52] Papargyropoulou E. Lozano R. Steinberger J. K. Wright N. Bin Ujang Z. (2014). The food waste hierarchy as a framework for the management of food surplus and food waste. J. Clean. Prod. 76, 106–115. doi: 10.1016/j.jclepro.2014.04.020

[ref53] Pedersen T. B. Ristagno D. McSweeney P. L. H. Vogensen F. K. Ardo Y. (2013). Potential impact on cheese flavour of heterofermentative bacteria from starter cultures. Int. Dairy J. 33, 112–119. doi: 10.1016/j.idairyj.2013.03.003

[ref54] Ren H. Feng Y. Liu T. Li J. Wang Z. Fu S.. (2019). Effects of different simulated seasonal temperatures on the fermentation characteristics and microbial community diversities of the maize straw and cabbage waste co-ensiling system. Sci. Total Environ. 708:135113. doi: 10.1016/j.scitotenv.2019.135113, PMID: 31791754

[ref55] Ren H. W. Tang D. L. Pei J. W. Fan W. G. Wang Z. Y. Peng Z. P.. (2019). Effects of three microbial inoculation on mixed storage quality and microbial community of dried corn straw and cabbage waste. J. Biobaased Mater. Bioenergy 13, 790–798. doi: 10.1166/jbmb.2019.1915

[ref56] Ren H. W. Wang C. Fan W. G. Zhang B. Y. Li Z. Z. Li D. (2018). Effects of formic or acetic acid on the storage quality of mixed air-dried corn stover and cabbage waste, and microbial community analysis. Food Technol. Biotechnol. 56, 71–82. doi: 10.17113/ftb.56.01.18.5455, PMID: 29795999 PMC5956268

[ref57] Restelatto R. Novinski C. O. Pereira L. M. Silva E. P. A. Volpi D. Zopollatto M.. (2019). Chemical composition, fermentative losses, and microbial counts of total mixed ration silages inoculated with different *Lactobacillus* species. J. Anim. Sci. 97, 1634–1644. doi: 10.1093/jas/skz030, PMID: 30715358 PMC6447279

[ref58] Robinson P. H. Wiseman J. Udén P. Mateos G. (2006). Some experimental design and statistical criteria for analysis of studies in manuscripts submitted for consideration for publication. Anim. Feed Sci. Technol. 129, 1–11. doi: 10.1016/j.anifeedsci.2006.05.011

[ref59] Rosenfelder P. Eklund M. Mosenthin R. (2013). Nutritive value of wheat and wheat by-products in pig nutrition: a review. Anim. Feed Sci. Technol. 185, 107–125. doi: 10.1016/j.anifeedsci.2013.07.011

[ref60] Santos V. H. D. Campos T. L. R. Espuny M. de Oliveira O. J. (2022). Towards a green industry through cleaner production development. Environ. Sci. Pollut. Res. 29, 349–370. doi: 10.1007/s11356-021-16615-2, PMID: 34674126

[ref61] Shang L. Wu Y. Wei N. Yang F. Wang M. Zhang L.. (2021). Novel arginine end-tagging antimicrobial peptides to combat multidrug-resistant bacteria. ACS Appl. Mater. Interfaces 14, 245–258. doi: 10.1021/acsami.1c19305, PMID: 34964342

[ref62] Sharif M. K. Butt M. S. Anjum F. M. Khan S. H. (2014). Rice bran: a novel functional ingredient. Crit. Rev. Food Sci. Nutr. 54, 807–816. doi: 10.1080/10408398.2011.608586, PMID: 24345050

[ref63] Shi B. M. He W. Su G. Xu X. D. Shan A. S. (2021). The effect of increasing neutral detergent fiber level through different fiber feed ingredients throughout the gestation of sows. Animals 11. doi: 10.3390/ani11020415, PMID: 33561988 PMC7914734

[ref64] Skrivanova E. Marounek M. (2007). Influence of pH on antimicrobial activity of organic acids against rabbit enteropathogenic strain of *Escherichia coli*. Folia Microbiol. 52, 70–72. doi: 10.1007/bf02932141, PMID: 17571799

[ref65] Van Soest P. J. Robertson J. B. Lewis B. A. (1991). Methods for dietary fiber, neutral detergent fiber, and nonstarch polysaccharides in relation to animal nutrition. J. Dairy Sci. 74, 3583–3597. doi: 10.3168/jds.S0022-0302(91)78551-2, PMID: 1660498

[ref66] Wang Y. He L. W. Xing Y. Q. Zheng Y. T. Zhou W. Pian R. Q.. (2019). Dynamics of bacterial community and fermentation quality during ensiling of wilted and unwilted *Moringa oleifera* leaf silage with or without lactic acid bacterial inoculants. mSphere 4:13. doi: 10.1128/mSphere.00341-19, PMID: 31391277 PMC6686226

[ref67] Wang Z. H. Li Q. K. Li J. Z. Shang L. Li J. W. Chou S. L.. (2022). pH-responsive antimicrobial peptide with selective killing activity for bacterial abscess therapy. J. Med. Chem. 65, 5355–5373. doi: 10.1021/acs.jmedchem.1c01485, PMID: 35294199

[ref68] Wang S. R. Yuan X. J. Dong Z. H. Li J. F. Shao T. (2017). Effect of ensiling corn stover with legume herbages in different proportions on fermentation characteristics, nutritive quality and in vitro digestibility on the Tibetan Plateau. Grassl. Sci. 63, 236–244. doi: 10.1111/grs.12173

[ref69] Wang S. Zhao J. Dong Z. Li J. Kaka N. A. Shao T. (2020). Sequencing and microbiota transplantation to determine the role of microbiota on the fermentation type of oat silage. Bioresour. Technol. 309:123371. doi: 10.1016/j.biortech.2020.123371, PMID: 32305853

[ref70] Yang L. L. Yuan X. J. Li J. F. Dong Z. H. Shao T. (2019). Dynamics of microbial community and fermentation quality during ensiling of sterile and nonsterile alfalfa with or without *Lactobacillus plantarum* inoculant. Bioresour. Technol. 275, 280–287. doi: 10.1016/j.biortech.2018.12.067 30594838

[ref71] Zhao X. Jiang L. Fang X. Y. Guo Z. Q. Wang X. X. Shi B. M.. (2022). Host-microbiota interaction-mediated resistance to inflammatory bowel disease in pigs. Microbiome 10:115. doi: 10.1186/s40168-022-01303-1, PMID: 35907917 PMC9338544

