# Supplementary material for: Anaerobic fermentation featuring wheat bran and rice bran realizes the clean transformation of Chinese cabbage waste into livestock feed
Source: Front Microbiol. 2023 Mar 24;14:1108047. doi: 10.3389/fmicb.2023.1108047 (PMC10079868; doi:10.3389/fmicb.2023.1108047)
Supplement: Supplementary file 1 [file Table_1.docx]

**Table S1** Fermentation characteristics of Chinese cabbage waste fermented alone or with wheat bran/rice bran.

| Treatments | Items | Groups | Days | | | | | | | Mean | SEM | Significance | | |
| --- | --- | --- | --- | --- | --- | --- | --- | --- | --- | --- | --- | --- | --- | --- |
|  |  |  | 1 | 3 | 5 | 7 | 15 | 30 | 60 |  |  | T | D | T×D |
| Wheat bran | Effluent (mL) | Con | 274.67±11.37^Aa^ | 306.00±8.00^ABa^ | 290.67±16.17^ABa^ | 319.33±16.17^Ba^ | 354.67±12.86^Ca^ | 377.33±3.06^Ca^ | 374.67±3.06^Ca^ | 328.19^a^ | 15.75 | * | * | * |
|  |  | W1 | 0^b^ | 0^b^ | 0^b^ | 0^b^ | 0^b^ | 0^b^ | 0^b^ | 0^b^ |  |  |  |  |
|  |  | W2 | 0^b^ | 0^b^ | 0^b^ | 0^b^ | 0^b^ | 0^b^ | 0^b^ | 0^b^ |  |  |  |  |
|  |  | W3 | 0^b^ | 0^b^ | 0^b^ | 0^b^ | 0^b^ | 0^b^ | 0^b^ | 0^b^ |  |  |  |  |
|  |  | Mean | 68.67^A^ | 76.50^BC^ | 72.67^AB^ | 79.83^C^ | 88.67^D^ | 94.3^D^ | 93.67^D^ |  |  |  |  |  |
|  | pH | Con | 4.68±0.02^Aa^ | 4.17±0.01^Ba^ | 3.95±0.02^Ca^ | 3.96±0.04^Ca^ | 3.96±0.01^Ca^ | 3.91±0.01^Ca^ | 3.79±0.02^Da^ | 4.06^a^ | 0.03 | * | * | * |
|  |  | W1 | 4.64±0.06^Aa^ | 4.19±0.02^Ba^ | 4.05±0.01^CDab^ | 4.08±0.01^Cb^ | 3.96±0.01^Da^ | 3.98±0.01^Db^ | 3.97±0.05^Db^ | 4.12^b^ |  |  |  |  |
|  |  | W2 | 4.87±0.06^Ab^ | 4.22±0.02^Bab^ | 4.11±0.01^Cb^ | 4.08±0.02^Cb^ | 3.91±0.01^Db^ | 3.93±0.02^Da^ | 4.01±0.01^Eb^ | 4.16^c^ |  |  |  |  |
|  |  | W3 | 4.98±0.06^Ac^ | 4.71±0.02^Bb^ | 4.23±0.08^Cc^ | 4.21±0.02^Cc^ | 4.06±0.03^Dc^ | 4.00±0.01^Db^ | 4.03±0.01^Db^ | 4.33^d^ |  |  |  |  |
|  |  | Mean | 4.81^A^ | 4.32^B^ | 4.09^C^ | 4.08^C^ | 3.97^D^ | 3.96^D^ | 3.95^D^ |  |  |  |  |  |
|  | Lactic acid (g/kg DM) | Con | 212.91±17.58^Aa^ | 266.71±3.62^Ba^ | 315.76±14.78^CDa^ | 318.23±4.52^CDa^ | 308.82±12.29^Ca^ | 328.65±24.16^CDa^ | 349.84±14.17^Da^ | 300.13^a^ | 9.71 | * | * | * |
|  |  | W1 | 55.87±18.21^Ab^ | 138.80±20.09^Bb^ | 129.56±31.55^Bb^ | 120.46±13.97^Bb^ | 200.09±14.12^Cb^ | 137.76±11.86^Bb^ | 158.89±6.30^BCb^ | 134.49^b^ |  |  |  |  |
|  |  | W2 | 50.96±1.11^Ab^ | 96.48±4.08^Bc^ | 123.91±11.90^BCb^ | 146.13±9.67^Cc^ | 146.61±25.96^Cc^ | 134.04±28.67^BCb^ | 146.55±6.29^Cb^ | 120.67^c^ |  |  |  |  |
|  |  | W3 | 42.31±2.29^Ab^ | 63.60±2.57^ABd^ | 91.40±35.09^BCb^ | 119.23±5.05^CDb^ | 136.54±20.59^Dc^ | 139.01±9.33^Db^ | 153.32±3.00^Db^ | 106.49^d^ |  |  |  |  |
|  |  | Mean | 90.51^A^ | 141.40^B^ | 165.16^C^ | 176.01^C^ | 198.02^D^ | 184.87^CD^ | 202.15^D^ |  |  |  |  |  |
|  | Acetic acid (g/kg DM) | Con | 6.94±1.42^Aa^ | 10.96±2.12^Aa^ | 13.16±2.19^Ba^ | 12.51±1.68^Ba^ | 13.07±2.29^Ba^ | 11.24±0.37^ABa^ | 12.51±0.84^Ba^ | 11.49^a^ | 0.42 | * | * | * |
|  |  | W1 | 3.04±1.26^Ab^ | 7.03±1.47^Bab^ | 7.97±2.17^Bb^ | 6.54±1.75^ABb^ | 12.49±0.33^Cab^ | 12.72±0.96^Ca^ | 16.45±1.29^Cb^ | 9.46^b^ |  |  |  |  |
|  |  | W2 | 3.84±0.66^Aab^ | 7.46±0.28^Bab^ | 8.84±1.00^BCb^ | 9.75±0.06^CEab^ | 9.87±0.44^CEbc^ | 12.44±0.51^Da^ | 10.92±0.97^DEa^ | 9.02^b^ |  |  |  |  |
|  |  | W3 | 3.87±0.37^Aab^ | 5.97±0.37^ABb^ | 6.78±0.97^BCb^ | 8.81±0.38^Cb^ | 7.65±0.33^Cc^ | 17.43±1.86^Db^ | 15.71±0.70^Db^ | 9.46^b^ |  |  |  |  |
|  |  | Mean | 4.42^A^ | 7.85^B^ | 9.19^BC^ | 9.40^CD^ | 10.77^D^ | 13.46^E^ | 13.90^E^ |  |  |  |  |  |
|  | Propionic acid (g/kg DM) | Con | ND | ND | ND | ND | ND | ND | ND | ND | 0.07 | NS | * | * |
|  |  | W1 | 2.45±0.25^a^ | 1.60±0.17^a^ | 2.25±0.06^a^ | 2.10±0.37^a^ | 1.98±0.80 | 2.53±1.01 | 2.06±0.25 | 2.14 |  |  |  |  |
|  |  | W2 | 2.38±0.19^a^ | 2.51±0.37^bc^ | 2.87±0.07^b^ | 2.11±0.12^a^ | 1.78±1.09 | 1.94±0.25 | 2.61±0.08 | 2.32 |  |  |  |  |
|  |  | W3 | 1.35±0.28^Ab^ | 1.99±0.32^ABac^ | 2.23±0.41^Ba^ | 1.37±0.18^Ab^ | 2.29±0.29^B^ | 3.37±0.04^C^ | 2.64±0.33^CB^ | 2.18 |  |  |  |  |
|  |  | Mean | 2.06^AB^ | 2.03^AB^ | 2.45^AB^ | 1.86^A^ | 2.02^AB^ | 2.62^B^ | 2.44^AB^ |  |  |  |  |  |
|  | WSC (g/kg DM) | Con | 82.69±0.71^Aa^ | 31.27±0.31^Ba^ | 22.67±1.00^Ca^ | 14.27±0.10^Da^ | 13.63±1.20^Da^ | 13.95±0.16^Da^ | 14.83±1.83^Da^ | 27.62^a^ | 1.81 | * | * | * |
|  |  | W1 | 67.66±2.41^Ab^ | 54.83±3.98^Bb^ | 49.61±3.15^BCb^ | 43.76±0.30^Cb^ | 32.38±1.71^Db^ | 31.94±0.49^Db^ | 31.96±0.39^Db^ | 44.59^b^ |  |  |  |  |
|  |  | W2 | 55.17±1.70^Ac^ | 45.62±1.60^Bc^ | 42.01±1.75^Bc^ | 41.47±2.59^Bb^ | 30.67±1.45^Cb^ | 28.15±1.01^Cc^ | 28.76±0.95^Cc^ | 38.84^c^ |  |  |  |  |
|  |  | W3 | 63.12±4.01^Ab^ | 55.92±3.20^Bb^ | 42.73±0.84^Cc^ | 43.34±0.76^Cb^ | 40.65±1.91^Cc^ | 40.64±1.89^Cd^ | 40.78±0.75^Cd^ | 46.74^d^ |  |  |  |  |
|  |  | Mean | 67.16^A^ | 46.91^B^ | 39.26^C^ | 35.71^D^ | 29.33^E^ | 28.67^E^ | 29.08^E^ |  |  |  |  |  |
| Rice bran | Effluent (mL) | Con | 274.67±11.37^Aa^ | 306.00±8.00^ABa^ | 290.67±16.17^ABa^ | 319.33±16.17^Ba^ | 354.67±12.86^Ca^ | 377.33±3.06^Ca^ | 374.67±3.06^Ca^ | 328.19^a^ | 15.75 | * | * | * |
|  |  | R1 | 0^b^ | 0^b^ | 0^b^ | 0^b^ | 0^b^ | 0^b^ | 0^b^ | 0^b^ |  |  |  |  |
|  |  | R2 | 0^b^ | 0^b^ | 0^b^ | 0^b^ | 0^b^ | 0^b^ | 0^b^ | 0^b^ |  |  |  |  |
|  |  | R3 | 0^b^ | 0^b^ | 0^b^ | 0^b^ | 0^b^ | 0^b^ | 0^b^ | 0^b^ |  |  |  |  |
|  |  | Mean | 68.67^A^ | 76.50^BC^ | 72.67^AB^ | 79.83^C^ | 88.67^D^ | 94.3^D^ | 93.67^D^ |  |  |  |  |  |
|  | pH | Con | 4.68±0.02^Aa^ | 4.17±0.01^Ba^ | 3.95±0.02^Ca^ | 3.96±0.04^Ca^ | 3.96±0.01^Ca^ | 3.91±0.01^Ca^ | 3.79±0.02^Da^ | 4.06^a^ | 0.03 | * | * | * |
|  |  | R1 | 4.95±0.03^Ab^ | 4.26±0.01^Bb^ | 4.10±0.01^Cb^ | 4.09±0.02^Cb^ | 4.06±0.01^Cb^ | 4.08±0.03^Cb^ | 4.09±0.02^Cb^ | 4.23^b^ |  |  |  |  |
|  |  | R2 | 4.89±0.05^Ab^ | 4.35±0.05^Bc^ | 4.24±0.03^Cc^ | 4.10±0.01^Db^ | 4.12±0.03^Dc^ | 4.14±0.02^Db^ | 4.15±0.04^CDbc^ | 4.28^c^ |  |  |  |  |
|  |  | R3 | 4.97±0.10^Ab^ | 4.54±0.04^Bd^ | 4.32±0.02^CEd^ | 4.22±0.05^CDc^ | 4.15±0.03^Dc^ | 4.14±0.02^Db^ | 4.22±0.02^DEc^ | 4.34^d^ |  |  |  |  |
|  |  | Mean | 4.87^A^ | 4.33^B^ | 4.16^C^ | 4.09^D^ | 4.07^D^ | 4.07^D^ | 4.06^D^ |  |  |  |  |  |
|  | Lactic acid (g/kg DM) | Con | 212.91±17.58^Aa^ | 266.71±3.62^Ba^ | 315.76±14.78^CDa^ | 318.23±4.52^CDa^ | 308.82±12.29^Ca^ | 328.65±24.16^CDa^ | 349.84±14.17^Da^ | 300.13^a^ | 11.23 | * | * | * |
|  |  | R1 | 38.90±5.26^Ab^ | 84.36±10.15^BCb^ | 81.45±11.77^BCb^ | 78.129±2.69^BCb^ | 70.03±9.30^Bb^ | 91.25±4.99^Cb^ | 87.17±2.25^BCb^ | 75.90^b^ |  |  |  |  |
|  |  | R2 | 47.38±1.66^Ab^ | 63.86±15.82^ABbc^ | 79.16±1.67^BCb^ | 65.52±11.10^ABb^ | 76.18±6.66^BCb^ | 101.78±12.55^Cb^ | 95.48±3.94^Cb^ | 75.62^b^ |  |  |  |  |
|  |  | R3 | 35.16±12.45^Ab^ | 51.89±12.63^ABb^ | 69.90±2.89^BCb^ | 70.60±4.05^BCb^ | 71.16±12.57^BCb^ | 81.67±7.52^Cb^ | 79.58±1.67^Cb^ | 65.71^c^ |  |  |  |  |
|  |  | Mean | 83.59^A^ | 116.71^B^ | 136.57^C^ | 133.12^C^ | 131.54^C^ | 150.84^D^ | 153.02^D^ |  |  |  |  |  |
|  | Acetic acid (g/kg DM) | Con | 6.94±1.42^Aa^ | 10.96±2.12^Aa^ | 13.16±2.19^Ba^ | 12.51±1.68^Ba^ | 13.07±2.29^Ba^ | 11.24±0.37^AB^ | 12.51±0.84^Ba^ | 11.49^a^ | 0.40 | * | * | * |
|  |  | R1 | 3.36±1.41^Ab^ | 4.75±1.93^Ab^ | 5.13±0.72^Ab^ | 5.46±0.45^Ab^ | 6.88±0.72^Ab^ | 13.12±1.21^B^ | 11.18±1.93^Bab^ | 7.13^b^ |  |  |  |  |
|  |  | R2 | 3.38±0.87^Ab^ | 4.50±1.59^Ab^ | 6.13±0.83^ABb^ | 5.66±0.33^ABb^ | 7.91±0.73^BCb^ | 9.88±1.70^C^ | 8.15±0.44^BCb^ | 6.52^b^ |  |  |  |  |
|  |  | R3 | 3.10±1.88^Ab^ | 4.25±1.28^ABb^ | 6.36±1.22^ABb^ | 5.15±1.86^ABb^ | 8.02±1.05^BCb^ | 10.34±1.41^CD^ | 13.43±0.76^Da^ | 7.24^b^ |  |  |  |  |
|  |  | Mean | 4.20^A^ | 6.11^B^ | 7.69^BC^ | 7.20^B^ | 8.97^C^ | 11.14^D^ | 11.32^D^ |  |  |  |  |  |
|  | WSC (g/kg DM) | Con | 82.69±0.71^Aa^ | 31.27±0.31^Ba^ | 22.67±1.00^Ca^ | 14.27±0.10^Da^ | 13.63±1.20^Da^ | 13.95±0.16^Da^ | 14.83±1.83^Da^ | 27.62^a^ | 1.55 | * | * | * |
|  |  | R1 | 14.48±0.57^Ab^ | 11.84±1.05^Bb^ | 10.18±0.66^Cb^ | 10.47±0.45^BCbc^ | 8.25±0.10^Dbc^ | 8.57±0.36^Dbc^ | 7.37±0.19^Db^ | 10.16^b^ |  |  |  |  |
|  |  | R2 | 14.75±1.12^Ab^ | 14.49±0.44^Ab^ | 10.83±0.12^Bb^ | 11.08±0.56^Bb^ | 11.40±0.68^Bb^ | 10.24±0.48^Bb^ | 7.45±0.80^Cb^ | 11.46^c^ |  |  |  |  |
|  |  | R3 | 18.153±1.33^Ab^ | 13.71±0.21^Bb^ | 9.19±0.83^Cb^ | 7.54±0.53^CDc^ | 6.40±0.57^DEc^ | 6.92±0.53^Dc^ | 4.54±0.44^Ec^ | 9.49^d^ |  |  |  |  |
|  |  | Mean | 32.52^A^ | 17.83^B^ | 13.22^C^ | 10.84^D^ | 9.92^D^ | 9.92^D^ | 8.55^E^ |  |  |  |  |  |

The significant difference (*p*<0.05) between different days (row) in the same group is represented by the different capital letters; The significant difference (*p*<0.05) between different groups (column) on the same day is represented by the different lowercase letters; The control group (Con). Chinese cabbage waste was mixed with wheat bran at a mass ratio of 383:117 (W1), 353:147 (W2), and 323:177 (W3) or with rice bran at 387:113 (R1), 358:142 (R2), and 329:171 (R3), respectively; WSC, water-soluble carbohydrate; DM, dry matter; FW, fresh weight; ND means not detected; T, the wheat bran/rice bran treatment; D, time duration; T×D, the interaction between the bran treatment and time duration; *, *p*<0.05; NS, *p*>0.05; SEM, standard error of means.
