# Supplementary material for: Anaerobic fermentation featuring wheat bran and rice bran realizes the clean transformation of Chinese cabbage waste into livestock feed
Source: Front Microbiol. 2023 Mar 24;14:1108047. doi: 10.3389/fmicb.2023.1108047 (PMC10079868; doi:10.3389/fmicb.2023.1108047)
Supplement: Supplementary file 2 [file Table_2.DOCX]

**Table S2** Microbial profiles of Chinese cabbage waste fermented alone or with wheat bran/rice bran.

| Treatments | Items | Groups | Days | | | | | | | Mean | SEM | Significance | | |
| --- | --- | --- | --- | --- | --- | --- | --- | --- | --- | --- | --- | --- | --- | --- |
|  |  |  | 1 | 3 | 5 | 7 | 15 | 30 | 60 |  |  | T | D | T×D |
| Wheat bran | LAB (log_10_ CFU/g FW) | Con | 5.82±0.07^Aa^ | 6.98±0.03^Ba^ | 5.70±0.31^ACa^ | 5.28±0.02^CDa^ | 5.23±0.17^Da^ | 5.11±0.13^Da^ | 5.05±0.19^Da^ | 5.60^a^ | 0.13 | * | * | * |
|  |  | W1 | 7.97±0.12^Ab^ | 7.91±0.09^Abc^ | 7.87±0.05^Ab^ | 8.08±0.07^Abc^ | 6.56±0.19^Bb^ | 6.17±0.06^Cb^ | 5.98±0.03^Cb^ | 7.22^b^ |  |  |  |  |
|  |  | W2 | 7.97±0.14^Ab^ | 7.66±0.12^Ab^ | 8.06±0.06^Ab^ | 7.89±0.08^Ab^ | 6.3±0.25^Bb^ | 5.72±0.15^Cab^ | 5.15±0.11^Da^ | 6.96^c^ |  |  |  |  |
|  |  | W3 | 8.04±0.07^Ab^ | 8.44±0.13^Bc^ | 8.12±0.08^Ab^ | 8.18±0.13^ABc^ | 7.40±0.03^Cc^ | 6.23±0.16^Db^ | 5.61±0.14^Eab^ | 7.43^d^ |  |  |  |  |
|  |  | Mean | 7.45^A^ | 7.75^B^ | 7.44^A^ | 7.36^A^ | 6.37^C^ | 5.81^D^ | 5.45^E^ |  |  |  |  |  |
|  | Coliform bacteria (log_10_ CFU/g FW) | Con | 6.54±0.20^Aa^ | 5.53±0.32^Ba^ | 5.04±0.15^BCab^ | 4.97±0.12^BCa^ | 4.65±0.30^CDab^ | 4.24±0.11^D^ | 5.28±0.11^Ba^ | 5.18^a^ | 0.08 | * | * | * |
|  |  | W1 | 5.45±0.22^Abc^ | 5.19±0.05^Aa^ | 5.01±0.03^ABab^ | 4.59±0.23^BDab^ | 3.98±0.33^Ca^ | 4.02±0.04^C^ | 4.21±0.06^CDb^ | 4.64^b^ |  |  |  |  |
|  |  | W2 | 6.21±0.36^Aad^ | 4.64±0.20^Bb^ | 4.50±0.22^BCa^ | 4.19±0.17^BCb^ | 3.96±0.36^BCa^ | 3.85±0.26^C^ | 4.24±0.19^BCb^ | 4.51^b^ |  |  |  |  |
|  |  | W3 | 5.78±0.29^Acd^ | 5.08±0.12^BCab^ | 5.36±0.40^ABCb^ | 5.63±0.22^ABc^ | 4.82±0.08^CEb^ | 3.75±0.06^D^ | 4.24±0.06^DEb^ | 4.95^c^ |  |  |  |  |
|  |  | Mean | 6.00^A^ | 5.11^B^ | 4.98^B^ | 4.84^B^ | 4.35^C^ | 3.97^D^ | 4.49^C^ |  |  |  |  |  |
|  | Yeast (log_10_ CFU/g FW) | Con | 5.37±0.34^AC^ | 5.28±0.23^ACa^ | 4.64±0.10^BCa^ | 4.43±0.05^Bab^ | 4.84±0.26^ABCa^ | 5.22±0.19^Ca^ | 4.58±0.22^Ba^ | 3.32^a^ | 0.07 | * | * | * |
|  |  | W1 | 4.93±0.33^A^ | 4.31±0.24^Bb^ | 4.27±0.05^Bb^ | 4.06±0.14^Ba^ | 4.22±0.33^Bb^ | 4.25±0.07^Bb^ | 4.16±0.09^Bb^ | 2.63^b^ |  |  |  |  |
|  |  | W2 | 5.24±0.19^A^ | 4.15±0.13^Bb^ | 4.12±0.15^Bb^ | 4.13±0.16^Ba^ | 4.25±0.20^Bab^ | 4.1±0.04^Bb^ | 4.12±0.01^Bb^ | 2.82^c^ |  |  |  |  |
|  |  | W3 | 4.93±0.10^A^ | 4.07±0.20^Bb^ | 4.37±0.05^BCb^ | 4.62±0.37^ACDb^ | 5.08±0.23^ADc^ | 4.11±0.02^BCb^ | 4.07±0.04^Bb^ | 3.19^a^ |  |  |  |  |
|  |  | Mean | 3.60^A^ | 2.81^BCD^ | 3.03^BE^ | 2.78^CD^ | 2.97^BCE^ | 2.60^D^ | 3.16^E^ |  |  |  |  |  |
| Rice bran | LAB (log_10_ CFU/g FW) | Con | 5.82±0.07^Aa^ | 6.98±0.03^Ba^ | 5.70±0.31^ACa^ | 5.28±0.02^CDa^ | 5.23±0.17^Da^ | 5.11±0.13^Da^ | 5.05±0.19^D^ | 5.60^a^ | 0.11 | * | * | * |
|  |  | R1 | 7.05±0.35^Ab^ | 6.91±0.39^Aa^ | 5.84±0.02^BEab^ | 5.26±0.04^BCa^ | 4.69±0.08^Ca^ | 4.63±0.32^Cb^ | 5.95±0.02^E^ | 5.76^a^ |  |  |  |  |
|  |  | R2 | 6.97±0.13^Ab^ | 8.79±0.10^Bb^ | 6.38±0.26^Cc^ | 5.85±0.15^Db^ | 5.54±0.25^Db^ | 5.90±0.19^CDc^ | 5.76±0.11^D^ | 6.45^b^ |  |  |  |  |
|  |  | R3 | 7.05±0.07^Ab^ | 8.71±0.24^Bb^ | 6.2±0.097^Cbc^ | 6.67±0.03^CDb^ | 5.50±0.35^Db^ | 5.92±0.40^CDc^ | 5.83±0.04^CD^ | 6.45^b^ |  |  |  |  |
|  |  | Mean | 6.72^A^ | 7.85^B^ | 6.05^C^ | 5.56^D^ | 5.24^E^ | 5.39^DE^ | 5.65^D^ |  |  |  |  |  |
|  | Coliform bacteria (log_10_ CFU/g FW) | Con | 6.54±0.20^Aa^ | 5.53±0.32^Ba^ | 5.04±0.15^BCa^ | 4.97±0.12^BCa^ | 4.65±0.30^CDa^ | 4.24±0.11^Da^ | 5.28±0.11^Ba^ | 5.18^a^ | 0.07 | * | * | * |
|  |  | R1 | 5.45±0.13^Ab^ | 4.99±0.11^ABb^ | 5.09±0.11^ABab^ | 5.34±0.18^ABb^ | 4.97±0.22^Ba^ | 4.08±0.26^Cab^ | 4.43±0.06^Cb^ | 4.91^b^ |  |  |  |  |
|  |  | R2 | 5.26±0.24^ACb^ | 4.86±0.14^ABb^ | 4.82±0.09^Bab^ | 5.31±0.08^Cb^ | 4.85±0.19^ABab^ | 3.67±0.19^Db^ | 4.32±0.03^Eb^ | 4.73^c^ |  |  |  |  |
|  |  | R3 | 5.37±0.30^Ab^ | 4.71±0.24^ABb^ | 4.77±0.13^ABb^ | 5.14±0.36^ADab^ | 4.20±0.23^BCb^ | 3.93±0.31^Cab^ | 4.44±0.07^BCDb^ | 4.65^c^ |  |  |  |  |
|  |  | Mean | 5.66^A^ | 5.02^BC^ | 4.93^B^ | 5.19^C^ | 4.67^D^ | 3.98^E^ | 4.62^D^ |  |  |  |  |  |
|  | Yeast (log_10_ CFU/g FW) | Con | 5.37±0.34^ACa^ | 5.28±0.23^ACa^ | 4.64±0.10^BCab^ | 4.43±0.05^B^ | 4.84±0.26^ABC^ | 5.22±0.19^Ca^ | 4.58±0.22^Ba^ | 3.32^a^ | 0.06 | * | * | * |
|  |  | R1 | 4.59±0.11^ACab^ | 4.63±0.12^ACb^ | 4.57±0.13^ACb^ | 4.36±0.13^A^ | 4.70±0.21^AC^ | 3.92±0.03^Bb^ | 4.77±0.06^Ca^ | 3.09^b^ |  |  |  |  |
|  |  | R2 | 4.43±0.22^ABbc^ | 4.53±0.14^Ab^ | 4.31±0.04^ABCc^ | 4.17±0.03^BC^ | 4.47±0.10^AB^ | 4.03±0.09^Cb^ | 4.20±0.04^BCb^ | 2.83^c^ |  |  |  |  |
|  |  | R3 | 3.67±0.46^Ac^ | 4.56±0.04^Bb^ | 4.28±0.01^Bc^ | 4.20±0.09^B^ | 4.34±0.03^B^ | 4.12±0.03^ABb^ | 4.13±0.09^ABb^ | 2.86^c^ |  |  |  |  |
|  |  | Mean | 3.63^A^ | 2.86^B^ | 2.81^B^ | 2.93^B^ | 2.37^C^ | 3.01^B^ | 3.57^A^ |  |  |  |  |  |

The significant difference (*p*<0.05) between different days (row) in the same group is represented by the different capital letters; The significant difference (*p*<0.05) between different groups (column) on the same day is represented by the different lowercase letters; The control group (Con). Chinese cabbage waste was mixed with wheat bran at a mass ratio of 383:117 (W1), 353:147 (W2), and 323:177 (W3) or with rice bran at 387:113 (R1), 358:142 (R2), and 329:171 (R3), respectively; LAB, lactic acid bacteria; CFU, colony-forming unit; FW, fresh weight; ND means not detected; T, the wheat bran/rice bran treatment; D, time duration; T×D, the interaction between the bran treatment and time duration; *, *p*<0.05; NS, *p*>0.05; SEM, standard error of means.
