# Supplementary material for: Anaerobic fermentation featuring wheat bran and rice bran realizes the clean transformation of Chinese cabbage waste into livestock feed
Source: Front Microbiol. 2023 Mar 24;14:1108047. doi: 10.3389/fmicb.2023.1108047 (PMC10079868; doi:10.3389/fmicb.2023.1108047)
Supplement: Supplementary file 3 [file Table_3.DOCX]

**Table S3** Nitrogen distribution of Chinese cabbage waste fermented alone or with wheat bran/rice bran.

| Treatments | Items | Groups | Days | | | | | | | Mean | SEM | Significant | | |
| --- | --- | --- | --- | --- | --- | --- | --- | --- | --- | --- | --- | --- | --- | --- |
|  |  |  | 1 | 3 | 5 | 7 | 15 | 30 | 60 |  |  | T | D | T×D |
| Wheat bran | Crude protein (g/kg DM) | Con | 248.56±1.61^Aa^ | 241.34±1.99^Ba^ | 247.48±1.32^ABa^ | 249.37±0.39^Aa^ | 249.97±2.15^Aa^ | 250.87±4.36^Aa^ | 250.48±2.75^Aa^ | 248.30^a^ | 3.27 | * | * | * |
|  |  | W1 | 180.05±0.78^ABb^ | 176.33±2.55^Ab^ | 178.86±1.97^ABb^ | 179.45±1.13^ABb^ | 183.51±1.76^BCb^ | 185.94±0.86^Cb^ | 188.23±2.60^Cb^ | 181.77^b^ |  |  |  |  |
|  |  | W2 | 174.54±0.32^Ac^ | 172.75±0.69^Ab^ | 173.64±0.47^Ac^ | 180.19±0.64^Bb^ | 184.97±1.91^Cb^ | 187.10±0.29^Cb^ | 187.78±1.82^Cb^ | 180.14^c^ |  |  |  |  |
|  |  | W3 | 180.12±0.47^Ab^ | 175.97±0.24^Bb^ | 172.49±0.94^Cc^ | 175.11±0.32^Bc^ | 183.32±0.67^Db^ | 185.83±0.40^Eb^ | 184.28±0.53^Db^ | 179.59^c^ |  |  |  |  |
|  |  | Mean | 195.82^A^ | 191.59^B^ | 193.12^B^ | 196.03^C^ | 200.44^D^ | 202.44^DE^ | 202.69^E^ |  |  |  |  |  |
|  | Nonprotein-N (g/kg TN) | Con | 672.27±3.59^Aa^ | 692.98±4.99^ACa^ | 722.90±2.85^Ba^ | 733.97±9.38^Ba^ | 736.83±4.40^Ba^ | 732.03±5.06^Ba^ | 714.64±17.93^BCa^ | 715.09^a^ | 10.07 | * | * | * |
|  |  | W1 | 541.07±12.08^Ab^ | 599.05±9.08^Bb^ | 678.92±5.84^Cb^ | 724.23±4.14^Da^ | 760.18±8.18^Eb^ | 790.40±5.80^Fb^ | 806.54±3.32^Fb^ | 700.06^b^ |  |  |  |  |
|  |  | W2 | 489.14±8.45^Ac^ | 584.52±20.68^Bbc^ | 606.16±4.39^Bc^ | 667.86±8.59^Cb^ | 770.71±6.79^Db^ | 808.06±4.64^Ec^ | 813.80±3.21^Eb^ | 677.18^c^ |  |  |  |  |
|  |  | W3 | 501.42±1.26^Ac^ | 560.09±1.26^Bc^ | 609.95±1.00^Cc^ | 628.67±3.33^Cc^ | 675.32±2.62^Dc^ | 739.38±15.16^Ea^ | 775.27±4.75^Fc^ | 641.44^d^ |  |  |  |  |
|  |  | Mean | 550.97^A^ | 609.16^B^ | 654.48^C^ | 688.68^D^ | 735.76^E^ | 767.47^F^ | 777.56^F^ |  |  |  |  |  |
|  | Free amino acid-N (g/kg TN) | Con | 179.78±4.16^Aa^ | 242.87±7.12^Ba^ | 253.97±6.48^Ba^ | 222.60±0.55^Ca^ | 144.00±3.48^Da^ | 132.21±2.86^D^ | 77.42±0.81^Ea^ | 194.02^a^ | 5.28 | * | * | * |
|  |  | W1 | 93.84±2.96^Ab^ | 103.12±3.80^ACb^ | 124.98±4.71^Bb^ | 111.09±2.63^CDb^ | 119.48±4.89^BDb^ | 137.5±1.95^E^ | 152.60±1.33^Fb^ | 120.38^b^ |  |  |  |  |
|  |  | W2 | 73.86±5.05^Ac^ | 93.75±1.30^Bb^ | 112.62±2.54^Cb^ | 108.79±1.11^Cb^ | 111.47±3.14^Cbc^ | 144.71±5.74^D^ | 195.61±2.78^Ec^ | 120.12^b^ |  |  |  |  |
|  |  | W3 | 69.36±2.37^Ac^ | 82.42±0.37^Bc^ | 91.67±6.89^Bc^ | 92.99±3.14^Bc^ | 106.28±1.82^Cc^ | 142.35±4.01^D^ | 143.99±7.24^Db^ | 104.15^c^ |  |  |  |  |
|  |  | Mean | 104.21^A^ | 130.54^B^ | 145.81^C^ | 133.87^B^ | 120.31^D^ | 139.20^E^ | 168.73^F^ |  |  |  |  |  |
|  | Ammonia-N (g/kg TN) | Con | 48.39±1.78^Aa^ | 55.39±2.13^Aa^ | 73.93±1.88^Ba^ | 85.69±3.17^Ca^ | 106.43±1.52^Da^ | 111.26±1.96^DEa^ | 118.26±5.58^Ea^ | 85.62^a^ | 2.90 | * | * | * |
|  |  | W1 | 26.26±0.77^Ab^ | 46.95±6.14^Ba^ | 50.71±1.69^Bb^ | 52.11±1.64^CEb^ | 63.30±5.17^Cb^ | 61.45±3.95^CEb^ | 70.393±1.43^Cb^ | 53.02^b^ |  |  |  |  |
|  |  | W2 | 21.44±2.21^Ab^ | 25.15±2.65^Ab^ | 33.12±2.74^Bc^ | 47.50±2.93^Cb^ | 45.97±4.54^Cc^ | 68.20±0.61^Dc^ | 67.45±1.52^Dbc^ | 44.12^c^ |  |  |  |  |
|  |  | W3 | 13.41±0.30^Ac^ | 18.02±0.77^Bb^ | 40.38±0.85^Cbc^ | 37.33±2.85^Cc^ | 36.88±0.74^Cd^ | 54.36±0.56^Dd^ | 60.43±2.88^Ec^ | 37.26^d^ |  |  |  |  |
|  |  | Mean | 27.38^A^ | 36.38^B^ | 49.53^C^ | 55.66^D^ | 63.14^E^ | 73.82^F^ | 79.13^G^ |  |  |  |  |  |
| Rice bran | Crude protein (g/kg DM)^2^ | Con | 248.56±1.61^Aa^ | 241.3±1.99^Ba^ | 247.48±1.32^ABa^ | 249.37±0.39^Aa^ | 249.97±2.15^Aa^ | 250.87±4.36^Aa^ | 250.48±2.75^Aa^ | 248.30^a^ | 8.37 | * | * | * |
|  |  | R1 | 76.94±0.92^Ab^ | 76.84±0.44^Ab^ | 75.55±0.23^Ab^ | 77.51±0.35^Ab^ | 75.64±0.31^Ab^ | 83.09±0.70^Bb^ | 85.00±2.34^Bb^ | 78.65^b^ |  |  |  |  |
|  |  | R2 | 70.49±1.32^ABCc^ | 72.67±0.63^ABCc^ | 73.18±1.22^ACb^ | 71.67±0.89^ABCc^ | 69.80±0.53^Bc^ | 73.39±0.22^Cc^ | 73.59±2.18^Cc^ | 72.11^c^ |  |  |  |  |
|  |  | R3 | 66.34±0.17^ABd^ | 66.51±1.80^ABd^ | 65.38±0.96^ABc^ | 65.13±0.72^ABd^ | 63.57±2.05^Ad^ | 67.80±0.43^Bd^ | 75.03±1.07^Cc^ | 67.11^d^ |  |  |  |  |
|  |  | Mean | 115.58^A^ | 114.34^A^ | 115.39^A^ | 115.92^A^ | 114.74^A^ | 118.79^B^ | 121.03^C^ |  |  |  |  |  |
|  | Nonprotein-N (g/kg TN) | Con | 672.27±3.59^Aa^ | 692.98±4.99^ACa^ | 722.90±2.85^Ba^ | 733.97±9.38^Ba^ | 736.83±4.40^Ba^ | 732.03±5.06^Ba^ | 714.64±17.93^BCa^ | 715.09^a^ | 9.14 | * | * | * |
|  |  | R1 | 528.67±5.09^Ab^ | 549.42±6.75^Bb^ | 557.52±7.81^Bb^ | 579.69±2.43^Cb^ | 612.85±3.24^Db^ | 652.73±5.44^Eb^ | 660.59±2.08^Eb^ | 591.64^b^ |  |  |  |  |
|  |  | R2 | 471.23±5.34^Ac^ | 477.25±4.63^Ac^ | 542.29±6.69^Bc^ | 552.78±1.33^Bc^ | 576.34±5.11^Cc^ | 617.80±1.95^Dc^ | 623.02±4.38^Dc^ | 551.53^c^ |  |  |  |  |
|  |  | R3 | 453.6±4.22^Ac^ | 514.11±4.99^Bd^ | 521.71±1.96^Bd^ | 521.93±6.71^Bd^ | 568.66±6.38^Cc^ | 596.18±6.04^Dd^ | 616.07±5.76^Ec^ | 541.76^d^ |  |  |  |  |
|  |  | Mean | 531.46^A^ | 558.44^B^ | 586.11^C^ | 597.10^D^ | 623.67^E^ | 649.69^F^ | 653.58^F^ |  |  |  |  |  |
|  | Free amino acid-N (g/kg TN) | Con | 179.78±4.16^Aa^ | 242.87±7.12^Ba^ | 253.97±6.48^Ba^ | 222.60±0.55^Ca^ | 144.00±3.48^Da^ | 132.21±2.86^Da^ | 77.42±0.81^Ea^ | 194.02^a^ | 4.77 | * | * | * |
|  |  | R1 | 152.07±1.93^Ab^ | 138.56±2.55^Bb^ | 164.76±3.03^Cb^ | 103.66±2.24^Db^ | 103.47±1.03^Db^ | 108.91±5.60^Db^ | 88.97±5.49^Ea^ | 122.92^b^ |  |  |  |  |
|  |  | R2 | 150.33±3.20^Ab^ | 107.52±2.75^Bc^ | 144.58±6.53^Ac^ | 135.17±1.48^Cc^ | 135.85±3.57^Ca^ | 153.85±9.32^Ac^ | 111.17±4.03^Bb^ | 134.07^c^ |  |  |  |  |
|  |  | R3 | 134.52±1.66^Ac^ | 93.26±1.80^BDd^ | 135.29±1.35^Ac^ | 93.39±2.78^BDd^ | 106.72±4.86^Cb^ | 108.08±7.40^Cb^ | 86.78±5.36^Da^ | 108.29^d^ |  |  |  |  |
|  |  | Mean | 154.17^A^ | 145.55^B^ | 174.65^C^ | 138.71^D^ | 122.51^EF^ | 125.76^E^ | 117.41^F^ |  |  |  |  |  |
|  | Ammonia-N (g/kg TN) | Con | 48.39±1.78^Aa^ | 55.39±2.13^Aa^ | 73.93±1.88^Ba^ | 85.69±3.17^Ca^ | 106.43±1.52^Da^ | 111.26±1.96^DEa^ | 118.26±5.58^Ea^ | 65.62^a^ | 2.81 | * | * | * |
|  |  | R1 | 37.86±1.27^Ab^ | 44.70±3.04^Ab^ | 93.78±1.31^BCb^ | 90.61±5.71^Ba^ | 101.22±4.13^Ca^ | 103.26±1.58^Db^ | 124.22±1.79^Ea^ | 85.09^a^ |  |  |  |  |
|  |  | R2 | 38.90±3.18^Ab^ | 56.58±2.47^Bc^ | 72.53±11.74^CDa^ | 66.46±0.93^BCb^ | 68.57±1.31^BCb^ | 83.30±2.77^Dc^ | 106.52±1.13^Eb^ | 70.41^b^ |  |  |  |  |
|  |  | R3 | 30.87±2.81^Ac^ | 63.62±2.22^Bc^ | 75.79±3.63^CDa^ | 71.18±2.85^BCb^ | 75.20±1.86^CDb^ | 80.88±3.06^Dc^ | 111.57±3.97^Eb^ | 72.73^b^ |  |  |  |  |
|  |  | Mean | 39.00^A^ | 55.07^B^ | 79.01^C^ | 78.48^C^ | 87.85^D^ | 94.68^E^ | 115.14^F^ |  |  |  |  |  |

The significant difference (*p*<0.05) between different days (row) in the same group is represented by the different capital letters; The significant difference (*p*<0.05) between different groups (column) on the same day is represented by the different lowercase letters; The control group (Con). Chinese cabbage waste was mixed with wheat bran at a mass ratio of 383:117 (W1), 353:147 (W2), and 323:177 (W3) or with rice bran at 387:113 (R1), 358:142 (R2), and 329:171 (R3), respectively; ND means not detected; T, the wheat bran/rice bran treatment; D, time duration; T×D, the interaction between the bran treatment and time duration; *, *p*<0.05; NS, *p*>0.05; SEM, standard error of means.
