# Supplementary material for: Anaerobic fermentation featuring wheat bran and rice bran realizes the clean transformation of Chinese cabbage waste into livestock feed
Source: Front Microbiol. 2023 Mar 24;14:1108047. doi: 10.3389/fmicb.2023.1108047 (PMC10079868; doi:10.3389/fmicb.2023.1108047)
Supplement: Supplementary file 4 [file Table_4.DOCX]

**Table S4** Chemical composition and antioxidant activity Chinese cabbage waste fermented alone or with wheat bran/rice bran

| Treatments | Groups | DPPH  (mg of trolox equivalent/g DM) | ABTS  (mg of trolox equivalent/g DM) | FRAP  (mg of trolox equivalent/g DM) | NDF  (g/kg DM) | ADF  (g/kg DM) | Ash  (g/kg DM) |
| --- | --- | --- | --- | --- | --- | --- | --- |
| Wheat bran | Con | 2.68±0.19^A^ | 6.67±0.24^A^ | 3.61±0.12^A^ | 263.77±6.92^A^ | 251.62±7.10^A^ | 106.03±1.67^A^ |
|  | W1 | 4.31±0.14^B^ | 8.72±0.26^B^ | 3.81±0.11^A^ | 372.14±6.28^B^ | 180.15±8.71^B^ | 67.82±0.68^B^ |
|  | W2 | 4.21±0.17^B^ | 8.66±0.11^B^ | 3.47±0.15^AB^ | 429.62±13.87^C^ | 185.71±2.89^B^ | 67.75±0.58^BC^ |
|  | W3 | 2.06±0.18^C^ | 8.43±0.55^B^ | 3.16±0.19^B^ | 432.08±14.30^C^ | 211.17±4.11^C^ | 65.18±0.59^C^ |
|  | SEM | 0.30 | 0.27 | 0.08 | 20.75 | 8.64 | 5.12 |
|  | *p* value | <0.05 | <0.05 | <0.05 | <0.05 | <0.05 | <0.05 |
| Rice bran | Con | 2.68±0.19^A^ | 6.67±0.24 | 3.61±0.12^A^ | 263.77±6.92^A^ | 251.62±7.10^A^ | 106.03±1.67^A^ |
|  | R1 | 2.04±0.12^B^ | 6.73±0.40 | 2.59±0.14^B^ | 713.24±14.05^B^ | 486.95±3.96^B^ | 85.48±1.18^B^ |
|  | R2 | 1.80±0.11^B^ | 6.29±0.64 | 2.60±0.12^B^ | 722.38±4.54^B^ | 500.21±8.10^B^ | 85.15±0.29^B^ |
|  | R3 | 1.98±0.11^B^ | 6.17±0.46 | 2.73±0.16^B^ | 757.96±17.00^C^ | 530.24±11.42^C^ | 84.15±0.73^B^ |
|  | SEM | 0.11 | 0.13 | 0.13 | 61.30 | 33.58 | 2.77 |
|  | *p* value | <0.05 | 0.402 | <0.05 | <0.05 | <0.05 | <0.05 |

The significant difference (*p*<0.05) between different groups (column) is represented by the different capital letters. The control group (Con). Chinese cabbage waste was mixed with wheat bran at a mass ratio of 383:117 (W1), 353:147 (W2), and 323:177 (W3) or with rice bran at 387:113 (R1), 358:142 (R2), and 329:171 (R3), respectively; DPPH, free radical DPPH scavenging activity; ABTS, radical ABTS scavenging activity; FRAP, ferric reducing antioxidant power; NDF, neutral detergent fiber; ADF, acid detergent fiber; Ash, crude ash; SEM, standard error of means.
