# Supplementary figures and images for: Anaerobic fermentation featuring wheat bran and rice bran realizes the clean transformation of Chinese cabbage waste into livestock feed
Source: Front Microbiol. 2023 Mar 24;14:1108047. doi: 10.3389/fmicb.2023.1108047 (PMC10079868; doi:10.3389/fmicb.2023.1108047)

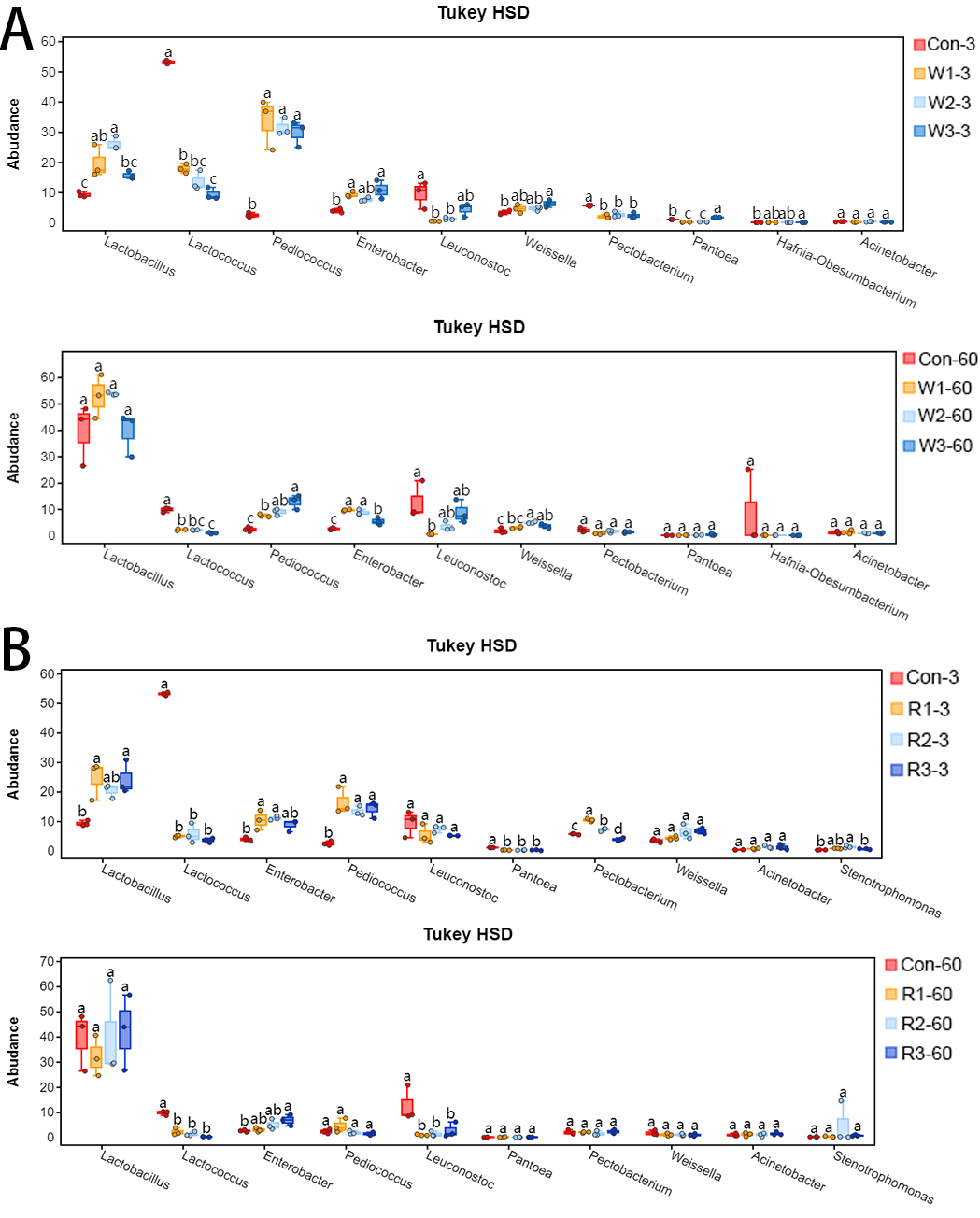

Supplement: Supplementary file 5 [file Image_1.TIF]

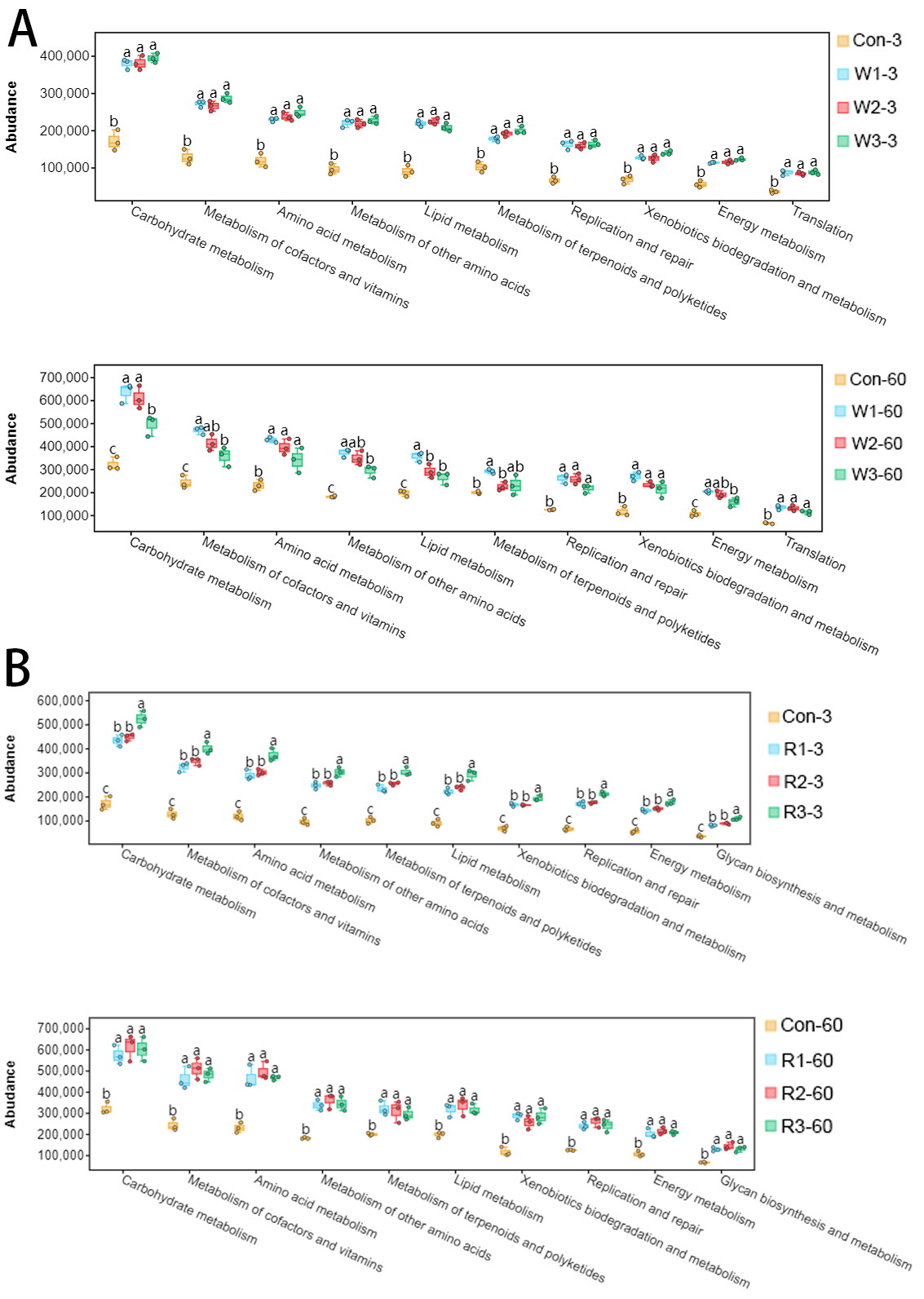

Supplement: Supplementary file 6 [file Image_2.TIF]

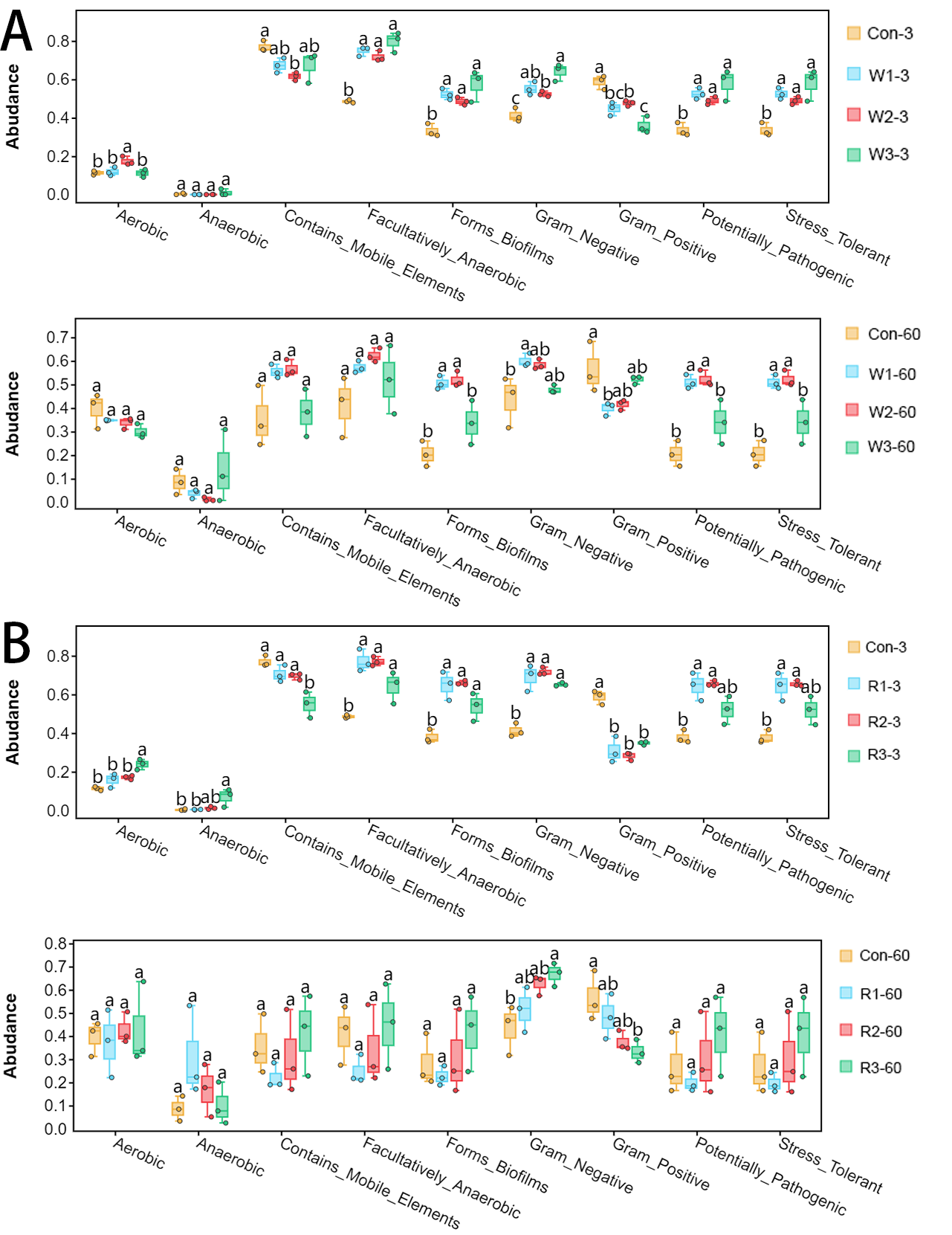

Supplement: Supplementary file 7 [file Image_3.TIF]
